# Supplementary material for: Theoretical Study on Vibrationally Resolved Electronic Spectra of Chiral Nanographenes
Source: Molecules. 2024 Aug 23;29(17):3999. doi: 10.3390/molecules29173999 (PMC11396777; doi:10.3390/molecules29173999)
Supplement: Supplementary file 1 [file molecules-29-03999-s001.zip › molecules-3170914-supplementary.pdf]

Theoretical Study on Vibrationally Resolved Electronic Spectra of Chiral Nanographenes

Yijian Ma, Xian Feng, Wenxiong Yu and Chengshuo Shen

Supplementary Materials 2
1. Benchmarks 2
2. Absorption and emission details 3
3. Hole-electron analysis 18
4. Selected vibrational modes 25
5. Geometries of S_0 and S_1 30
6. Spectrum of OO7H and OO9H 32
7. Cartesian coordinates of the optimized geometries (in Å) 34
8. Reference 54

Supplementary Materials

1. Benchmarks

In order to validate the accuracy of exchange-correlation functionals, we conducted a series of computational studies on the vibronic absorption and emission spectrum of **BP**. Employing CAM-B3LYP-D3(BJ) [1-3], PBE0-D3(BJ) [4], M06-2X-D3 [5], and ωB97XD [6] functionals, we meticulously optimized the ground and excited states (S_0 and S_1) of both compounds, utilizing the def-TZVP basis set [7]. Subsequently, Time-Dependent Density Functional Theory (TD-DFT) calculations were executed on the optimized structures, again applying the same suite of functionals but with the def2-TZVP basis set for enhanced precision. These comprehensive benchmark calculations were systematically carried out in the gas phase, leveraging the advanced capabilities of the Gaussian 09 program [8], to ensure that our results provide a robust foundation for understanding the electronic properties of these complex molecular systems.

The results demonstrate that the vibrationally resolved electron spectroscopy is not significantly influenced by different functionals; after undergoing a certain degree of redshift or blueshift, it aligns well with the experimental outcomes, showcasing a commendable correspondence.

Figure S1. Vibronic absorption and emission spectrum of **BP** for four different four functionals. Calculated using Gaussian 09 program with TD approach at 0K.

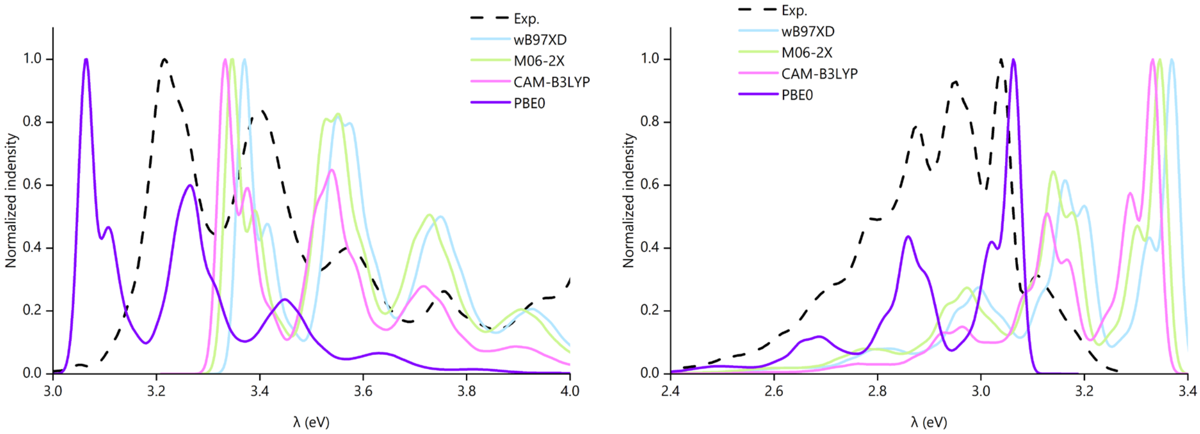

2. Absorption and emission details

Table S1. Selected dominant excitations and occupied (occ) – unoccupied (unocc) MO pair contributions (greater than 10%) of **5H** in the ground state S_0.

Excitation	*E* (eV)	λ (nm)	*f*	occ no.	unocc no.	%
1	3.4738	356.91	0.0011	73	74	59.8
				72	75	38.3
2	3.6956	335.49	0.0039	72	74	51.4
				73	75	47.0
3	3.9517	313.75	0.3469	73	75	45.3
				72	74	45.0
4	4.0979	302.56	0.0623	72	75	54.8
				73	74	35.9
5	4.1826	296.43	0.0186	71	74	61.1
				72	76	28.4

Table S2. Selected dominant excitations and occupied (occ) – unoccupied (unocc) MO pair contributions (greater than 10%) of **5H** in the ground state S_1.

Excitation	*E* (eV)	λ (nm)	*f*	occ no.	unocc no.	%
1	3.2089	386.38	0.0018	73	74	62.0
				72	75	36.4
2	3.4446	358.94	0.0021	72	74	50.2
				73	75	38.7
3	3.7328	332.15	0.3612	73	75	53.6
				72	74	36.6
4	3.8593	321.26	0.0724	72	75	58.4
				73	74	34.5
5	4.0179	308.58	0.0205	71	74	62.1
				72	76	29.4

Table S3. Selected dominant excitations and occupied (occ) – unoccupied (unocc) MO pair contributions (greater than 10%) of **6H** in the ground state S_0.

Excitation	*E* (eV)	*λ* (nm)	*f*	occ no.	unocc no.	%
1	3.3306	372.26	0.0027	85	87	54.3
				86	88	42.0
2	3.4823	356.04	0.0012	86	87	78.3
				85	88	20.3
3	3.7415	331.38	0.3101	86	88	54.6
				85	87	38.9
4	3.8914	318.61	0.0439	85	88	49.3
				83	88	20.2
				86	87	14.3
5	3.9756	311.86	0.0071	84	87	92.0

Table S4. Selected dominant excitations and occupied (occ) – unoccupied (unocc) MO pair contributions (greater than 10%) of **6H** in the ground state S_1.

Excitation	*E* (eV)	*λ* (nm)	*f*	occ no.	unocc no.	%
1	3.0814	402.36	0.0008	85	87	50.9
				86	88	46.5
2	3.2520	381.26	0.0006	86	88	77.8
				178	181	23.5
3	3.5233	351.90	0.3174	86	88	50.4
				85	87	43.7
4	3.6869	336.28	0.0718	85	88	64.4
				86	87	18.4
				83	87	10.9
5	3.8707	320.31	0.0049	84	87	92

Table S5. Selected dominant excitations and occupied (occ) – unoccupied (unocc) MO pair contributions (greater than 10%) of **7H** in the ground state S_0.

Excitation	*E* (eV)	λ (nm)	*f*	occ no.	unocc no.	%
1	3.1428	394.50	0.0003	99	100	59.7
				98	101	37.8
2	3.3004	375.66	0.0263	98	100	96.8
3	3.3740	367.47	0.0534	99	101	89.4
4	3.6114	343.31	0.0403	97	100	72.0
				98	102	12.2
5	3.6512	339.57	0.0124	98	101	53.7
				99	100	32.9

Table S6. Selected dominant excitations and occupied (occ) – unoccupied (unocc) MO pair contributions (greater than 10%) of **7H** in the ground state S_1.

Excitation	*E* (eV)	λ (nm)	*f*	occ no.	unocc no.	%
1	2.8732	431.52	0.0005	99	100	62.4
				98	101	35.9
2	3.0505	406.44	0.0269	98	100	97.5
3	3.1059	399.19	0.0513	99	101	94.9
4	3.4188	362.65	0.0268	98	101	60.5
				99	100	34.8
5	3.4894	355.32	0.0216	97	100	74.5
				98	102	13.5

Table S7. Selected dominant excitations and occupied (occ) – unoccupied (unocc) MO pair contributions (greater than 10%) of **BP** in the ground state S_0.

Excitation	*E* (eV)	*λ* (nm)	*f*	occ no.	unocc no.	%
1	3.3059	375.04	0.2170	72	73	94.6
2	3.4718	357.12	0.0003	71	73	49.6
				72	74	49.2
3	4.1092	301.72	0.0031	72	75	88.8
4	4.2600	291.04	0.3639	71	73	46.1
				72	74	46.1
5	4.3665	283.94	0.0094	70	73	68.7
				71	74	13.1
				72	76	10.4

Table S8. Selected dominant excitations and occupied (occ) – unoccupied (unocc) MO pair contributions (greater than 10%) of **BP** in the ground state S_0.

Excitation	*E* (eV)	*λ* (nm)	*f*	occ no.	unocc no.	%
1	2.9480	420.57	0.2474	72	73	96.7
2	3.3443	370.73	0.0003	71	73	49.9
				72	74	48.8
3	3.9024	317.71	0.0082	72	75	88.7
4	4.1491	298.82	0.3715	72	74	45.1
				71	73	45.1
5	4.1509	298.69	0.0049	70	73	79.9

Table S9. Selected dominant excitations and occupied (occ) – unoccupied (unocc) MO pair contributions (greater than 10%) of **O7H** in the ground state S_0.

Excitation	E (eV)	λ (nm)	f	occ no.	unocc no.	%
1	2.7633	448.68	0.1605	98	99	96.9
2	3.0526	406.16	0.0002	98	100	49.0
				97	99	48.7
3	3.5118	353.05	0.1205	97	99	36.3
				98	100	34.1
				96	99	23.2
4	3.5531	348.95	0.0149	98	101	51.9
				96	99	36.0
5	3.8949	318.32	0.1852	98	101	24.2
				96	99	22.0
				97	100	16.6

Table S10. Selected dominant excitations and occupied (occ) – unoccupied (unocc) MO pair contributions (greater than 10%) of **O7H** in the ground state S_1.

Excitation	E (eV)	λ (nm)	f	occ no.	unocc no.	%
1	2.2223	557.91	0.1302	98	99	97.8
2	2.8712	431.82	0.0001	98	100	50.3
				97	99	47.3
3	3.2551	380.89	0.0027	96	99	58.8
				98	101	33.9
4	3.3015	375.54	0.0658	98	100	36.5
				97	99	36.0
				98	101	21.0
5	3.6239	342.13	0.0410	98	102	77.1

Table S11. Selected dominant excitations and occupied (occ) – unoccupied (unocc) MO pair contributions (greater than 10%) of **O8H** in the ground state S_0.

Excitation	*E* (eV)	*λ* (nm)	*f*	occ no.	unocc no.	%
1	2.6685	464.62	0.1210	111	112	96.9
2	2.9919	414.40	0.0015	111	113	56.7
				110	112	37.2
3	3.3524	369.84	0.0107	109	112	83.5
4	3.3922	365.50	0.1405	110	112	36.7
				111	113	35.1
				111	114	17.8
5	3.5710	347.20	0.0245	108	112	48.5
				111	115	31.1

Table S12. Selected dominant excitations and occupied (occ) – unoccupied (unocc) MO pair contributions (greater than 10%) of **O8H** in the ground state S_1.

Excitation	*E* (eV)	*λ* (nm)	*f*	occ no.	unocc no.	%
1	2.2175	559.12	0.1263	111	112	98.1
2	2.8275	438.49	0.0007	111	113	58.1
				110	112	23.5
				109	112	15.9
3	3.0737	403.37	0.0276	110	112	40.4
				109	112	33.5
				111	114	21.8
4	3.2171	385.39	0.0301	109	112	46.4
				111	113	26.8
				111	114	16.1
5	3.3548	369.57	0.0194	108	112	52.0
				111	115	36.1

Table S13. Selected dominant excitations and occupied (occ) – unoccupied (unocc) MO pair contributions (greater than 10%) of **O9H** in the ground state S_0.

Excitation	*E* (eV)	*λ* (nm)	*f*	occ no.	unocc no.	%
1	2.5168	492.63	0.0777	124	125	95.1
2	2.8135	440.68	0.0005	124	126	49.9
				123	125	44.9
3	3.0778	402.83	0.0263	123	125	47.8
				124	126	36.6
4	3.2570	380.67	0.0382	122	125	59.9
				124	127	20.4
5	3.4317	361.29	0.0059	121	125	60.5
				124	128	16.9
				123	126	10.1

Table S14. Selected dominant excitations and occupied (occ) – unoccupied (unocc) MO pair contributions (greater than 10%) of **O9H** in the ground state S_1.

Excitation	*E* (eV)	*λ* (nm)	*f*	occ no.	unocc no.	%
1	1.8871	657.01	0.0495	124	125	96.9
2	2.5942	477.93	0.0006	124	126	53.2
				123	125	42.8
3	2.8881	429.29	0.0332	123	125	43.7
				124	126	30.1
				122	125	19.1
4	2.9580	419.15	0.0296	122	125	54.3
				124	127	24.2
5	3.1311	395.98	0.0017	121	125	58.1
				124	128	33.2

Table S15. Selected dominant excitations and occupied (occ) – unoccupied (unocc) MO pair contributions (greater than 10%) of **OO7H** in the ground state S_0.

Excitation	*E* (eV)	*λ* (nm)	*f*	occ no.	unocc no.	%
1	3.0283	409.42	0.1212	97	98	69.5
				96	98	13.0
2	3.0465	406.97	0.0354	97	99	42.2
				96	98	34.5
				97	98	20.0
3	3.6758	337.30	0.4127	96	98	33.8
				97	99	32.5
				97	100	27.5
4	3.8126	325.20	0.1911	97	100	63.8
				96	98	11.6
5	3.9484	314.01	0.0850	95	98	51.9
				96	99	24.1
				97	101	11.9

Table S16. Selected dominant excitations and occupied (occ) – unoccupied (unocc) MO pair contributions (greater than 10%) of **OO7H** in the ground state S_1.

Excitation	*E* (eV)	*λ* (nm)	*f*	occ no.	unocc no.	%
1	2.7978	443.15	0.1883	97	98	92.9
2	2.9291	423.28	0.0005	97	99	51.8
				96	98	46.6
3	3.5385	350.39	0.2363	97	100	53.4
				97	99	21.3
				96	98	21.2
4	3.6673	338.08	0.3200	97	100	36.1
				95	98	21.1
				96	98	20.5
				97	99	16.0
5	3.7931	326.87	0.1355	95	98	67.3

Table S17. Selected dominant excitations and occupied (occ) – unoccupied (unocc) MO pair contributions (greater than 10%) of **OO9H** in the ground state S_0.

Excitation	*E* (eV)	*λ* (nm)	*f*	occ no.	unocc no.	%
1	2.7782	446.28	0.2254	123	124	69.6
				123	125	13.3
2	2.7889	444.56	0.0702	122	124	41.3
				123	125	35.1
				123	124	19.9
3	3.2942	313.75	0.2069	123	125	45.1
				122	124	43.8
4	3.3758	302.56	0.0036	121	124	46.1
				123	126	44.6
5	3.5533	296.43	0.2876	122	125	52.6
				123	126	17.7
				121	124	17.6

Table S18. Selected dominant excitations and occupied (occ) – unoccupied (unocc) MO pair contributions (greater than 10%) of **OO9H** in the ground state S_1.

Excitation	*E* (eV)	*λ* (nm)	*f*	occ no.	unocc no.	%
1	2.4600	504.00	0.3913	123	124	94.3
2	2.6626	465.65	0.0004	122	124	50.9
				123	125	47.3
3	3.1541	393.09	0.0961	122	124	33.7
				123	125	31.6
				121	124	24.2
4	3.1872	389.01	0.0393	123	126	42.4
				121	124	29.4
				123	125	13.5
5	3.4347	360.98	0.0574	123	126	36.7
				121	124	34.2

Figure S2. Molecular orbitals of helicenes, **BP** and nanographenes.

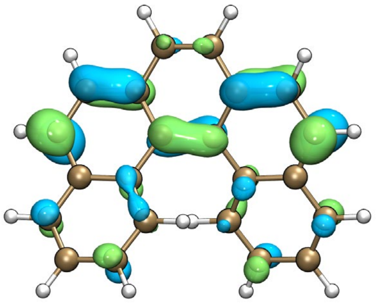

5H-MO 75
−1.494 eV

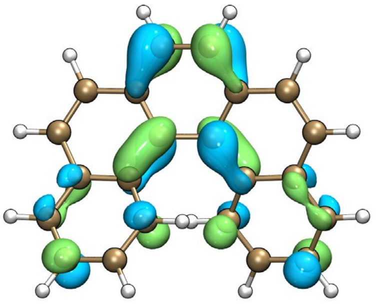

5H-MO 74
−1.530 eV

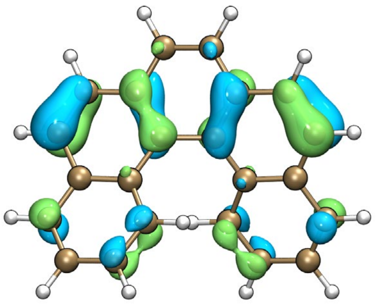

5H-MO 73
−6.010 eV

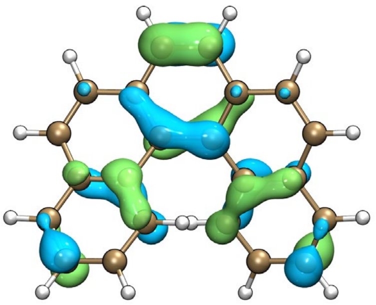

5H-MO 72
−6.135 eV

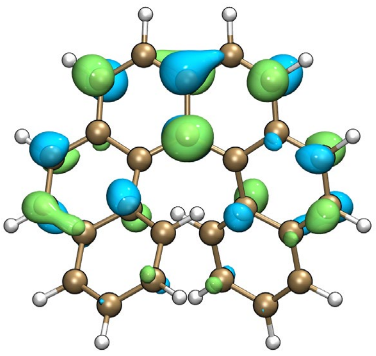

6H-MO 88
−1.504 eV

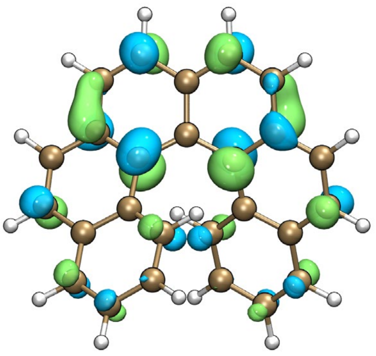

6H-MO 87
−1.694 eV

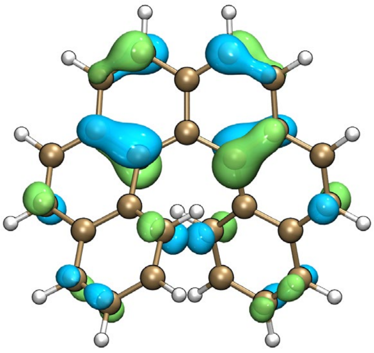

6H-MO 86
−5.943 eV

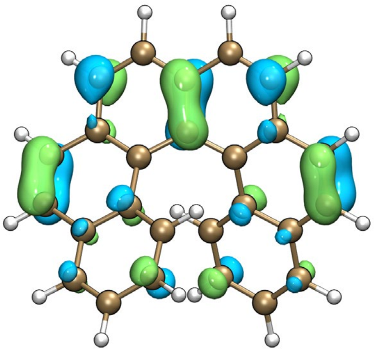

6H-MO 85
−6.016 eV

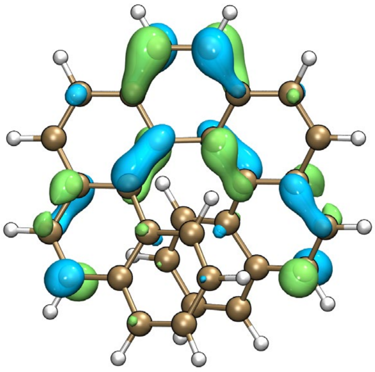

7H-MO 101
−1.659 eV

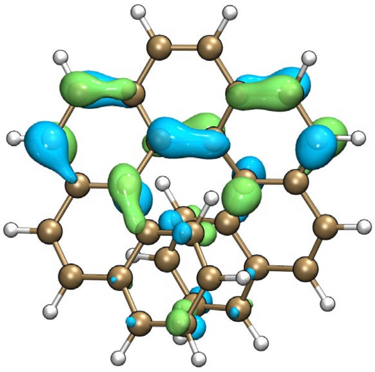

7H-MO 100
−1.737 eV

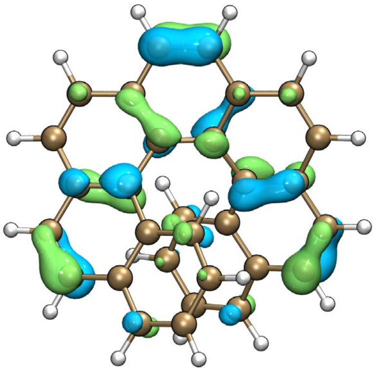

7H-MO 99
−5.860 eV

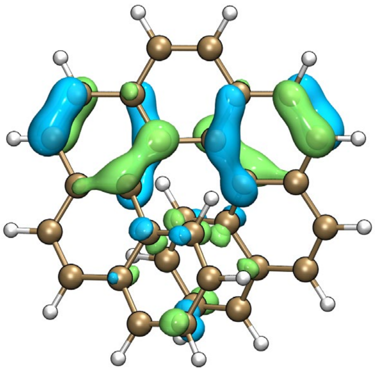

7H-MO 98
−5.918 eV

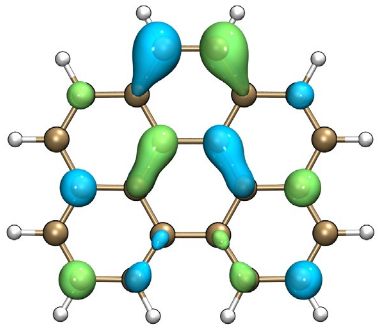

BP-MO 74
−1.158 eV

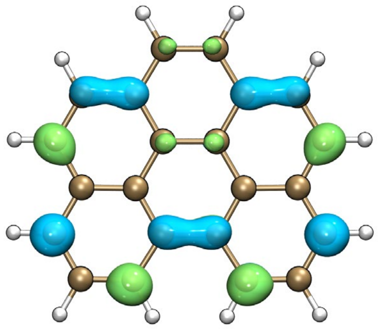

BP-MO 73
−1.874 eV

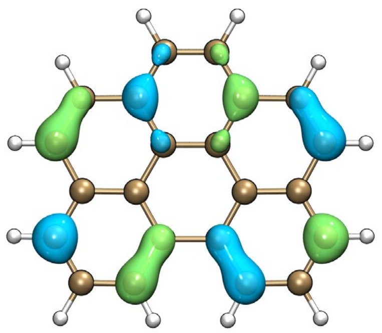

BP-MO 72
−5.685 eV

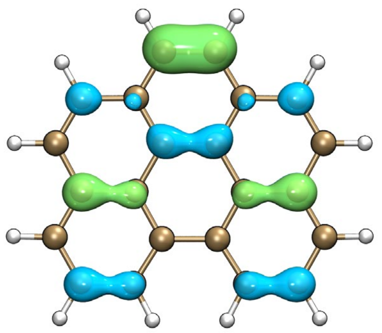

BP-MO 71
−6.372 eV

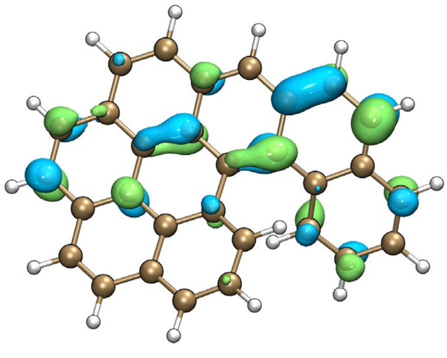

O7H-MO 100
−1.437 eV

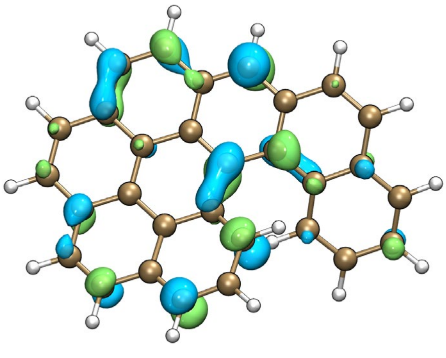

O7H-MO 99
−2.142 eV

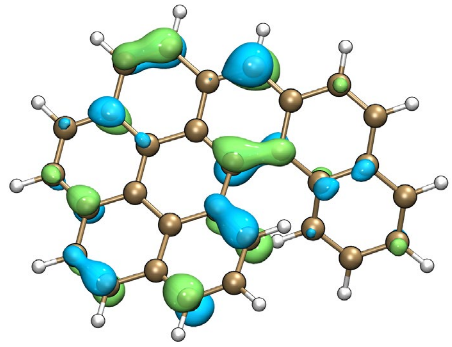

O7H-MO 98
−5.456 eV

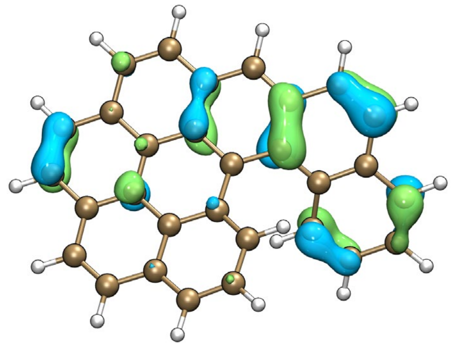

O7H-MO 97
−6.145 eV

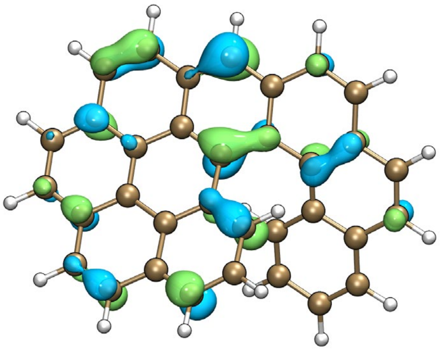

O8H-MO 113
−1.494 eV

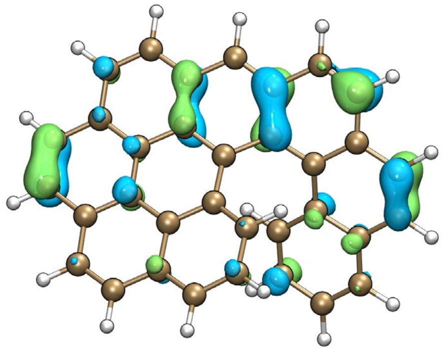

O8H-MO 112
−2.174 eV

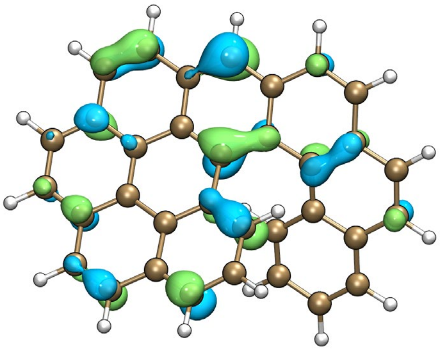

O8H-MO 111
−5.414 eV

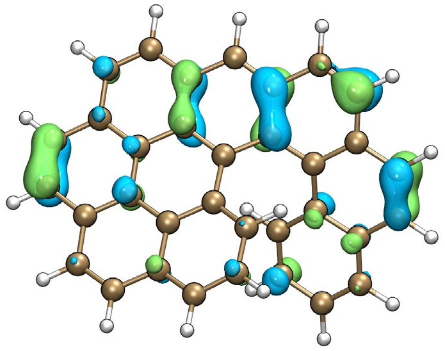

O8H-MO 110
−6.144 eV

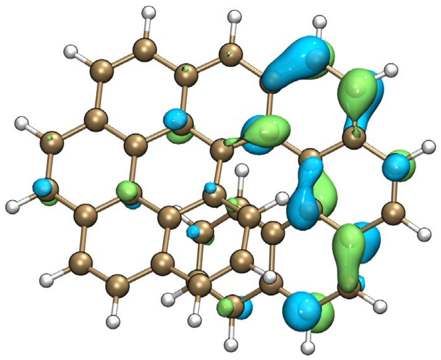

O9H-MO 126
−1.642 eV

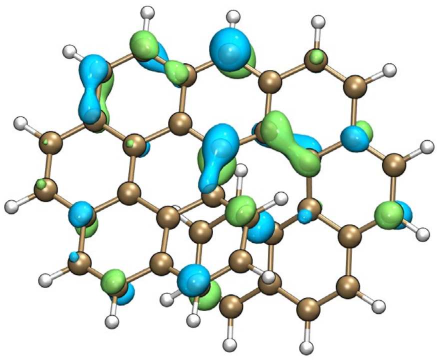

O9H-MO 125
−2.196 eV

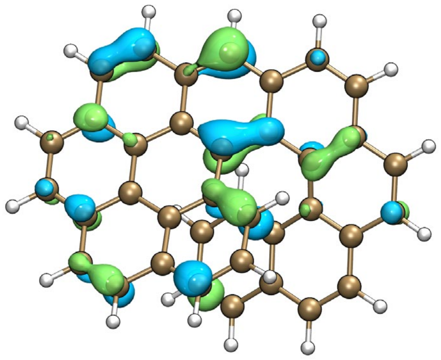

O9H-MO 124
−5.357 eV

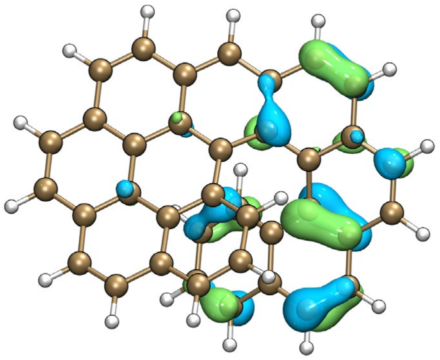

O9H-MO 123
−5.930 eV

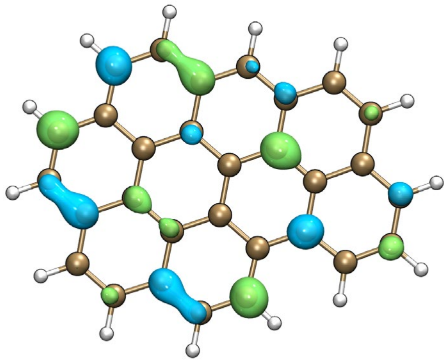

OO7H-MO 99
−1.573 eV

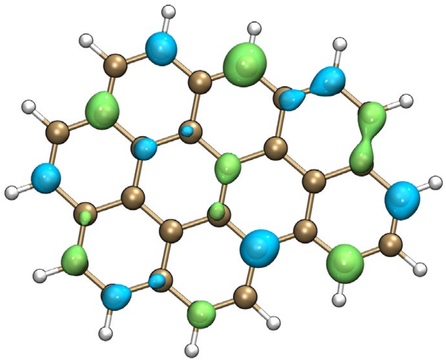

OO7H-MO 98
−2.038 eV

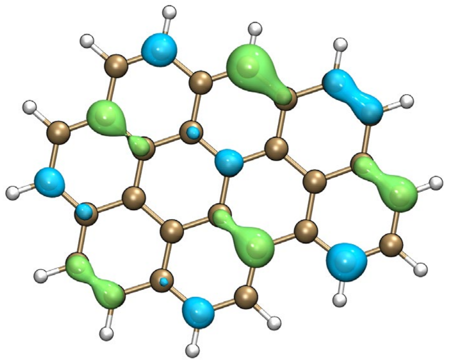

OO7H-MO 97
−5.537 eV

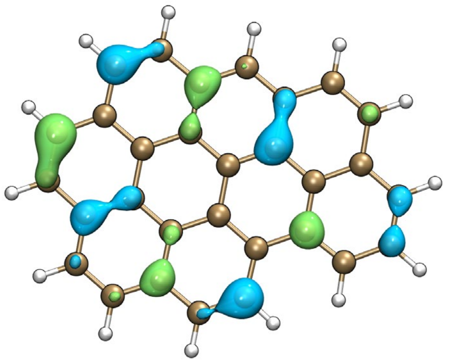

OO7H-MO 96
−6.020 eV

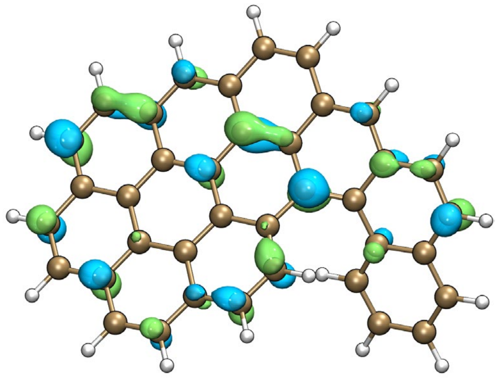

OO9H-MO 125
−1.490 eV

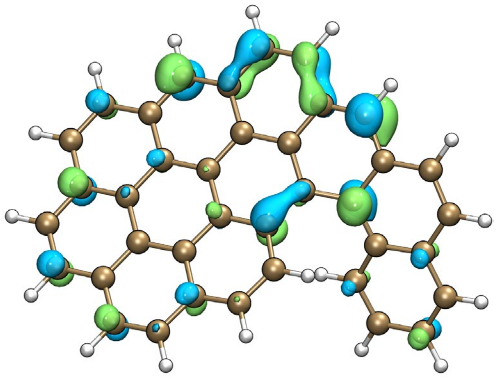

OO9H-MO 124
−2.092 eV

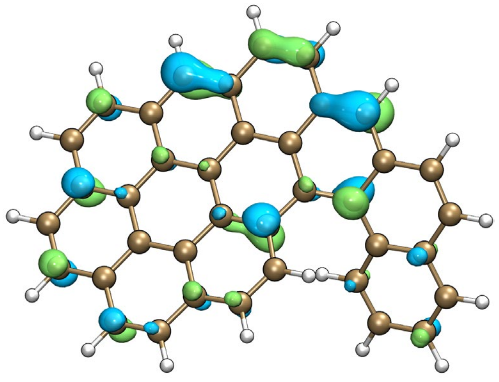

OO9H-MO 123
−5.446 eV

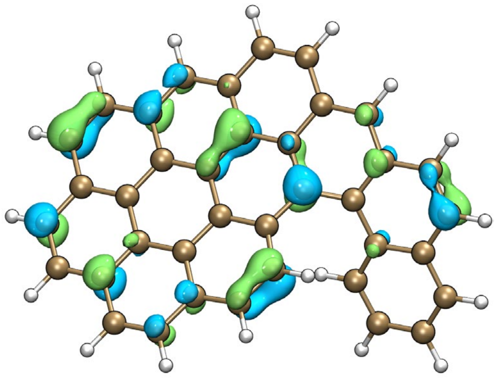

OO9H-MO 122
−5.846 eV

3. Hole-electron analysis

Figure S3. Hole-electron analysis of $S_0 \rightarrow S_1$ transition.

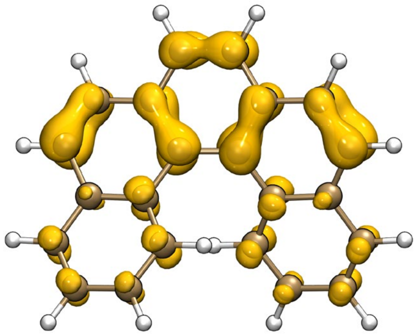

5H-hole

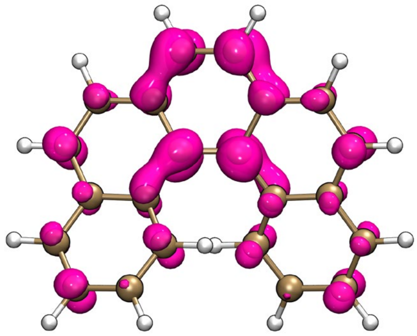

5H-electron

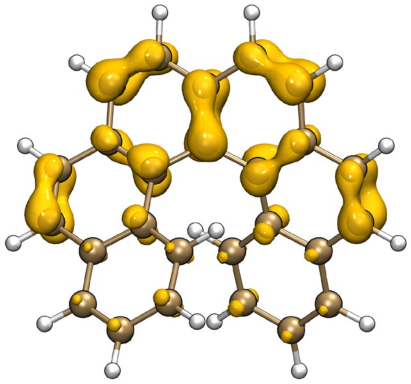

6H-hole

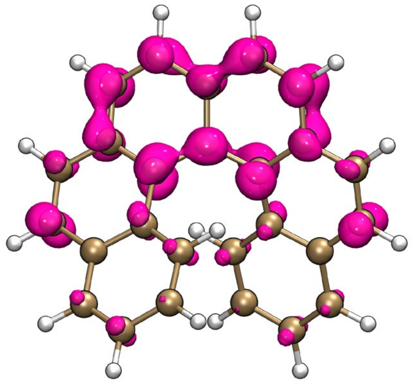

6H-electron

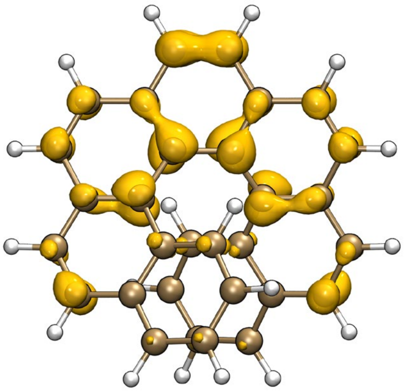

7H-hole

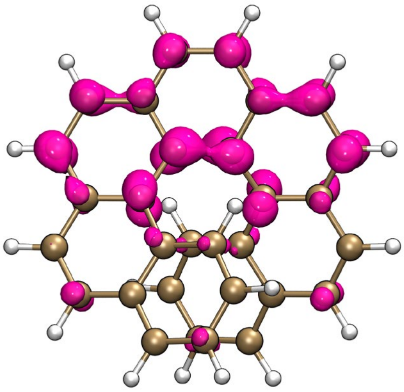

7H-electron

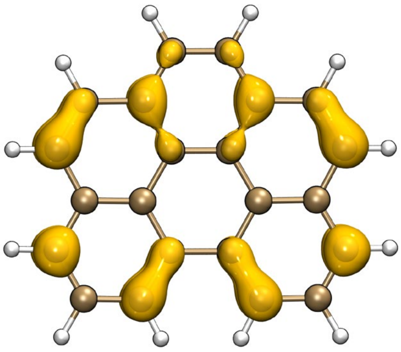

BP-hole

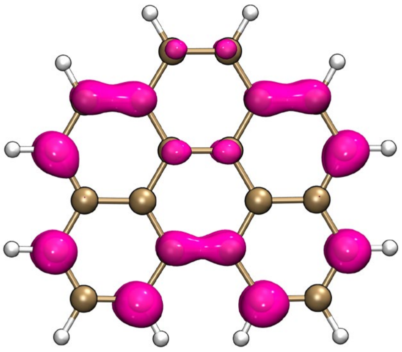

BP-electron

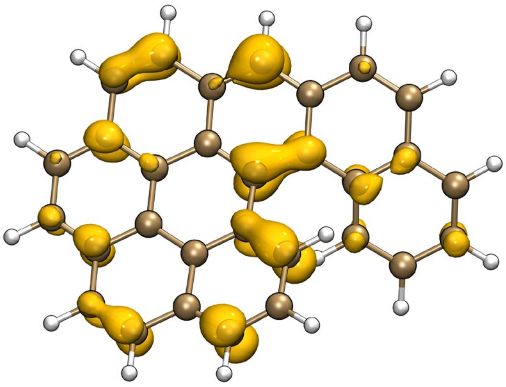

O7H-hole

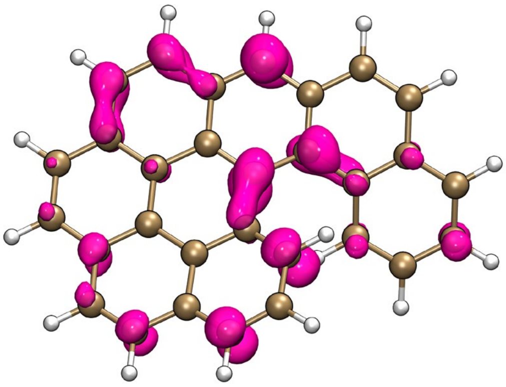

O7H-electron

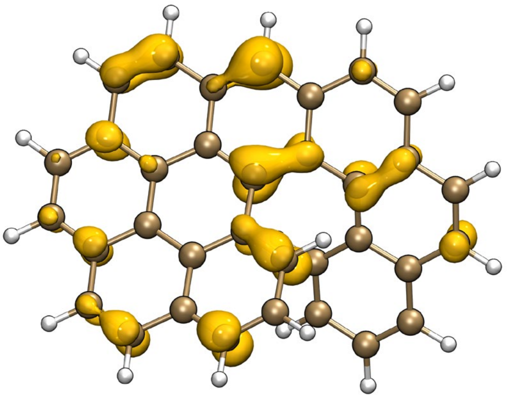

O8H-hole

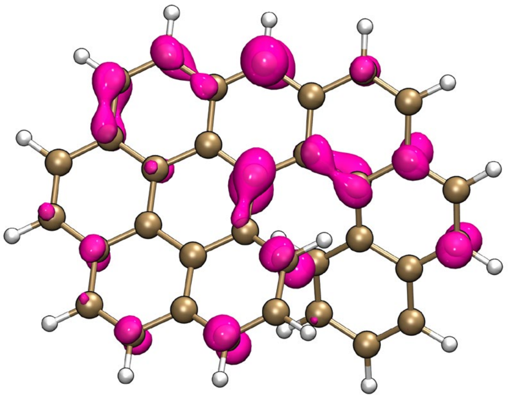

O8H-electron

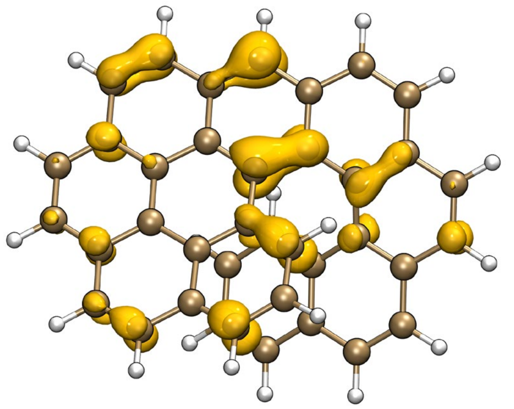

O9H-hole

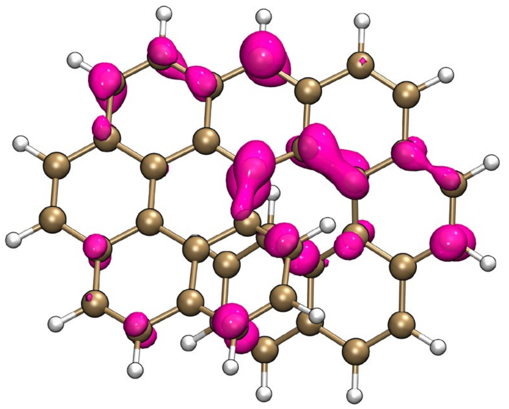

O9H-electron

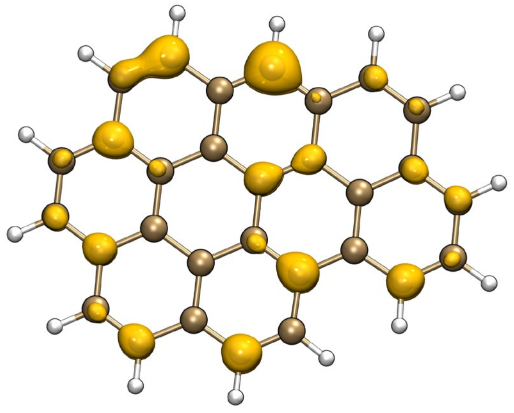

OO7H-hole

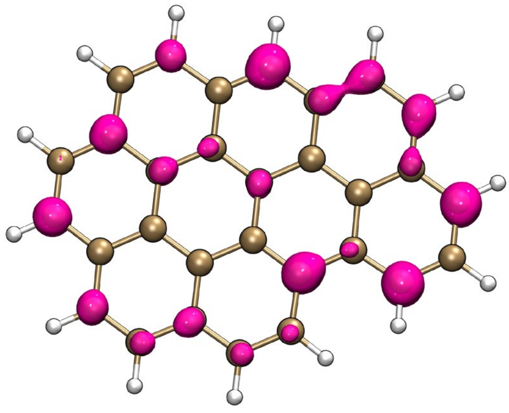

OO7H-electron

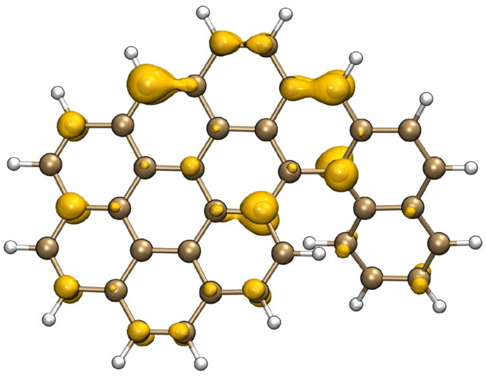

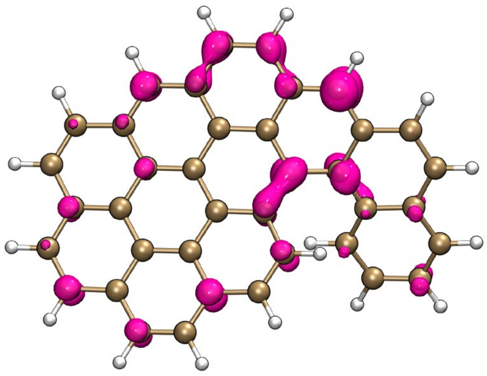

OO9H-hole **OO9H-electron**

Figure S4. Hole-electron analysis of $S_1 \rightarrow S_0$ transition.

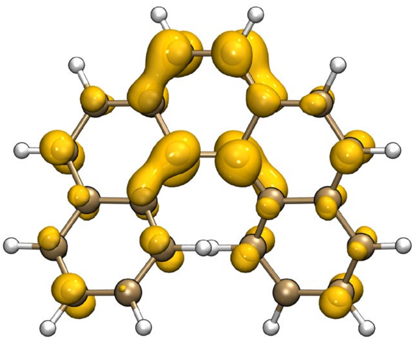

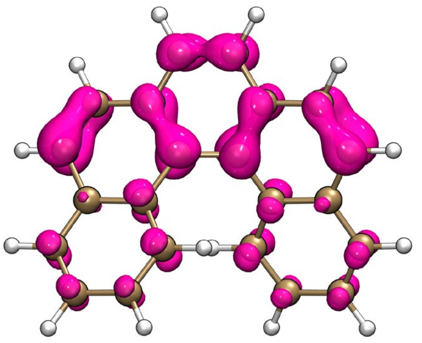

5H-hole **5H-electron**

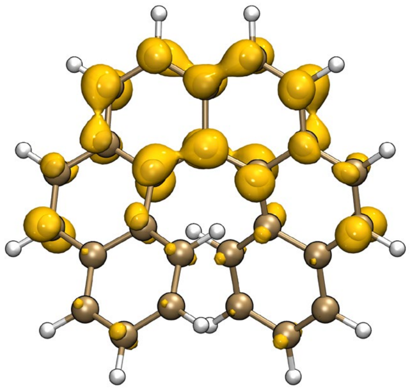

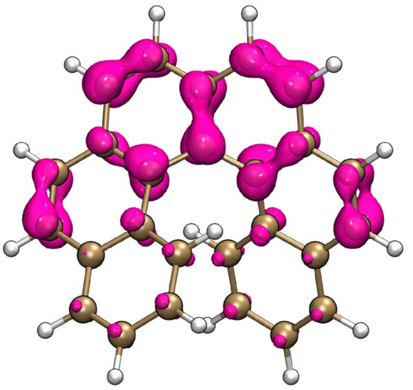

6H-hole **6H-electron**

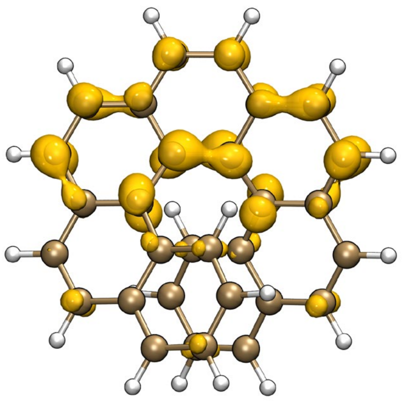

7H-hole

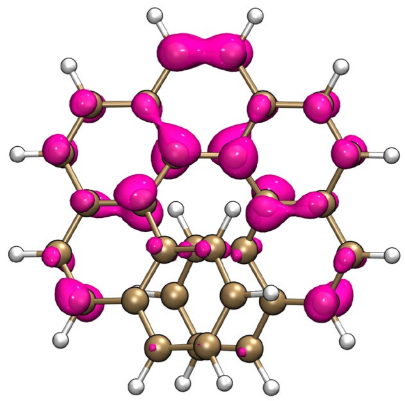

7H-electron

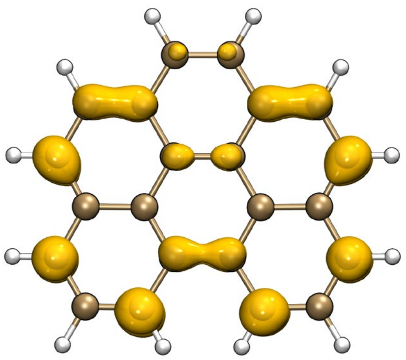

BP-hole

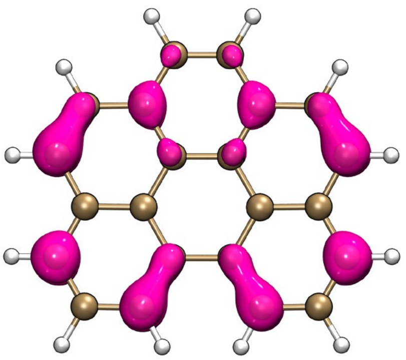

BP-electron

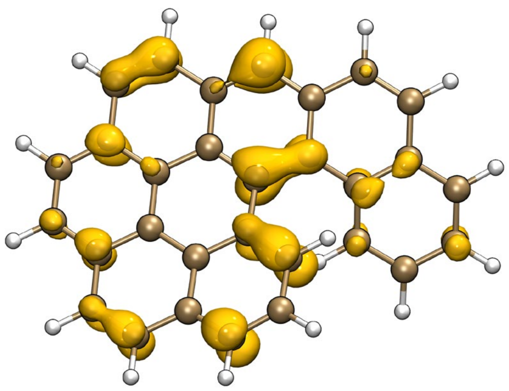

O7H-hole

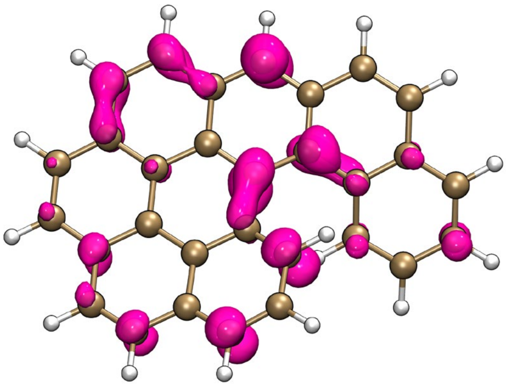

O7H-electron

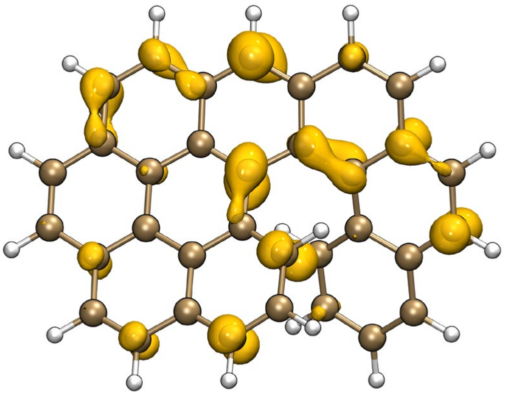

O8H-hole

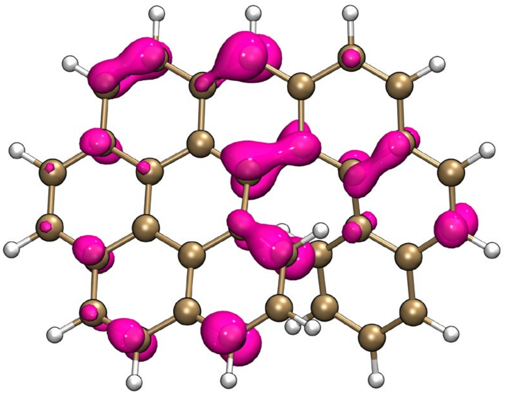

O8H-electron

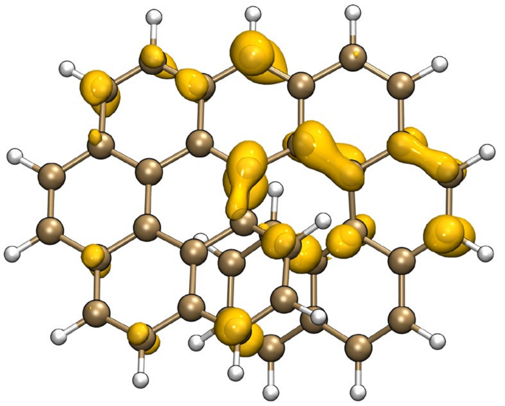

O9H-hole

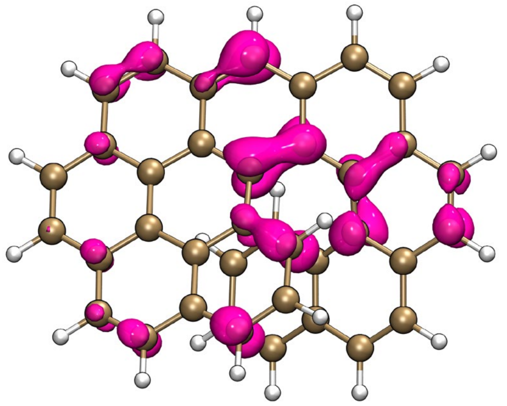

O9H-electron

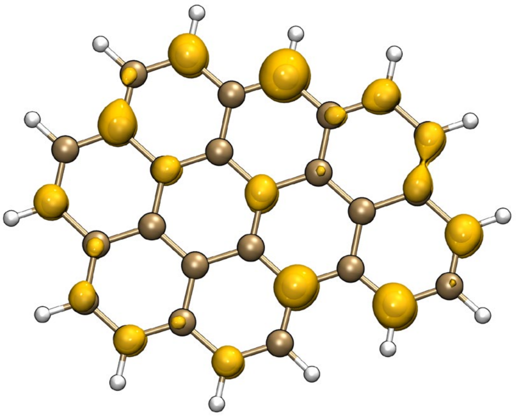

OO7H-hole

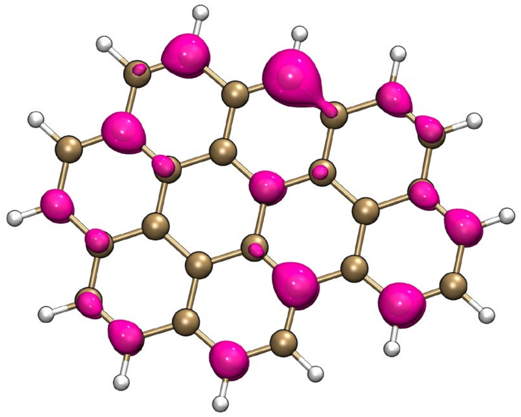

OO7H-electron

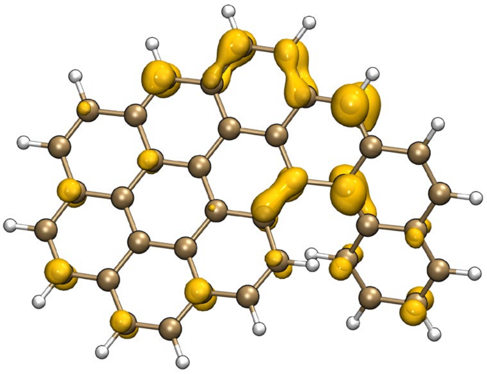

OO9H-hole

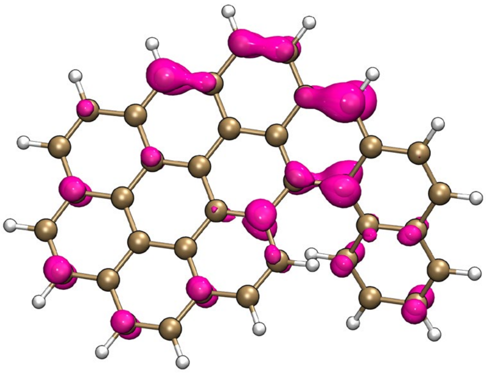

OO9H- electron

4. Selected vibrational modes

Figure S5. Selected vibrational modes of **O7H**, **O8H**, **O9H** and **OO9H** at S_0 and S_1 states with prominent relaxation energies (in cm^{-1}).

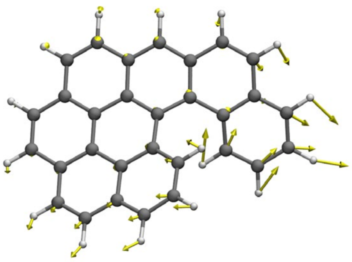

O7H-S_0-mode 3 (152)

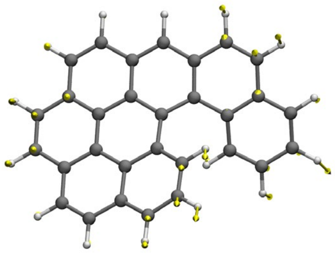

O7H-S_0-mode 5 (320)

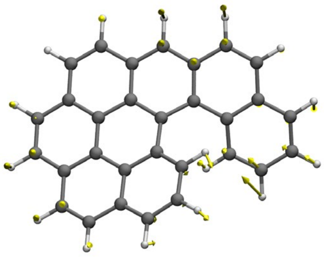

O7H-S_0-mode 6 (350)

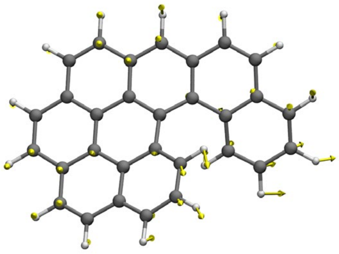

O7H-S_0-mode 9 (1475)

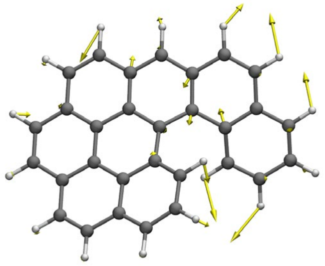

O7H-S_0-mode 88 (1638)

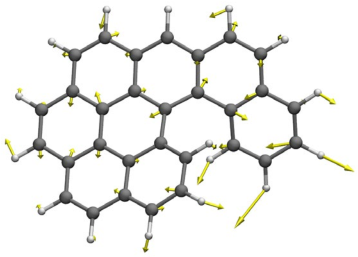

O7H-S_0-mode 108 (1677)

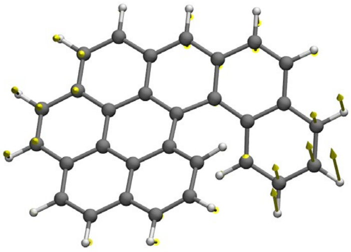

O7H-S_1-mode 1 (88)

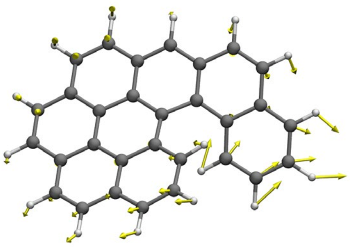

O7H-S_1-mode 3 (156)

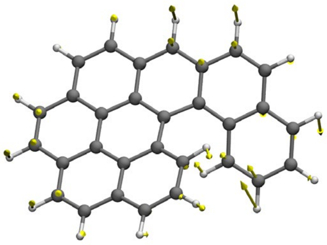

O7H-S_1-mode 6 (313)

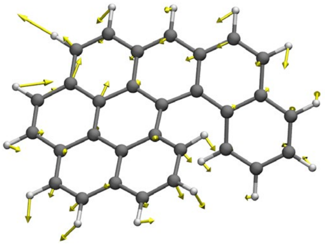

O7H-S_1-mode 22 (1448)

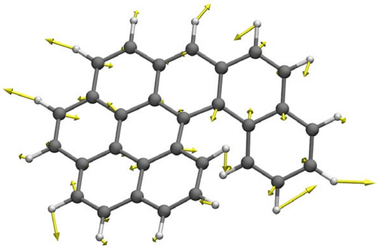

O7H-S_1-mode 103 (1645)

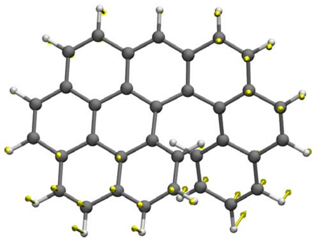

O8H-S_0-mode 1 (39)

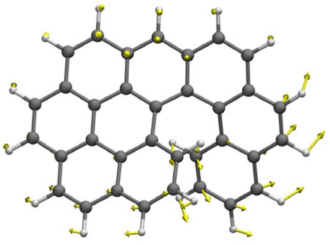

O8H-S_0-mode 3 (65)

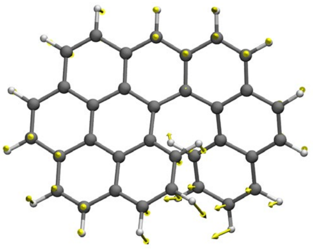

O8H-S_0-mode 4 (86)

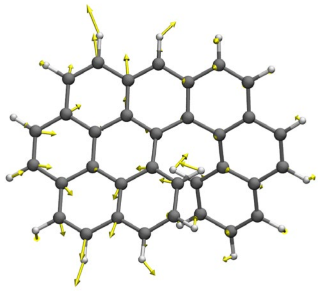

O8H-S_0-mode 20 (400)

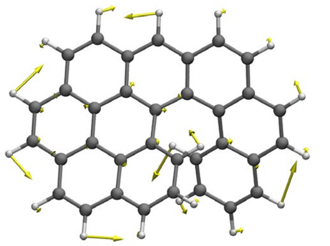

O8H-S_0-mode 99 (1292)

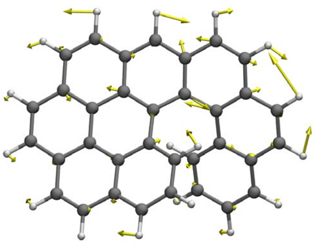

O8H-S_0-mode 108 (1427)

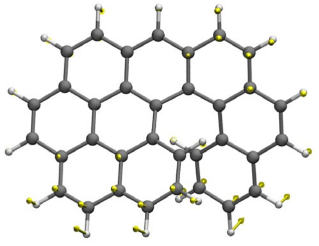

O8H-S_1-mode 1 (113)

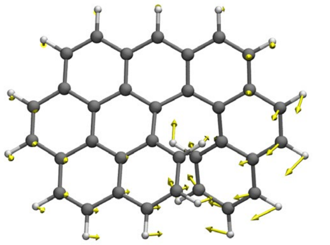

O8H-S_1-mode 3 (204)

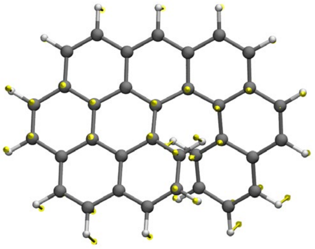

O8H-S_1-mode 5 (353)

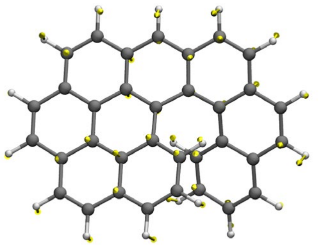

O8H-S_1-mode 13 (1496)

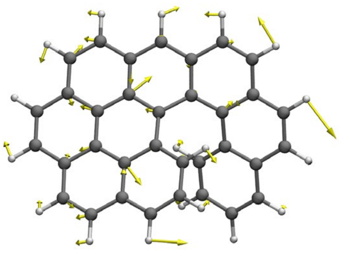

O8H-S_1-mode 108 (1651)

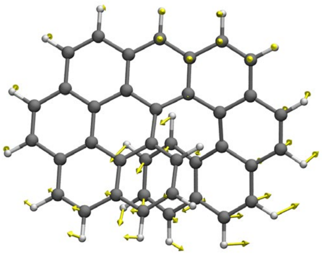

O9H-S_0-mode 3 (104)

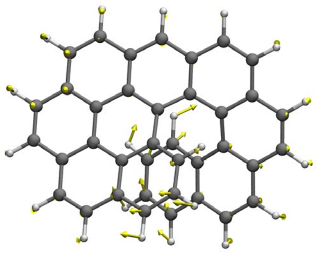

O9H-S_0-mode 7 (244)

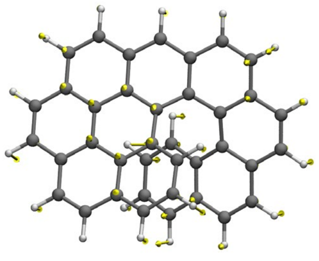

O9H-S_0-mode 9 (337)

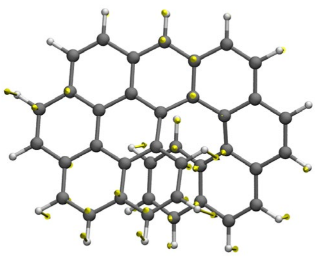

O9H-S_0-mode 13 (1431)

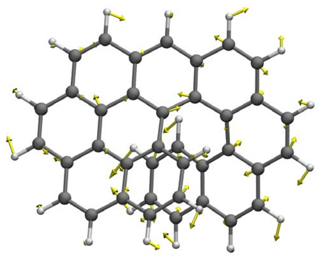

O9H-S_0-mode 138 (1656)

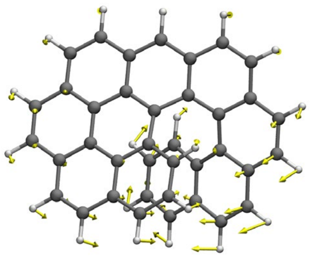

O9H-S_1-mode 3 (99)

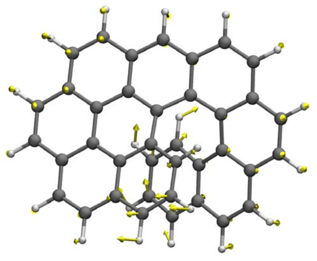

O9H-S_1-mode 7 (261)

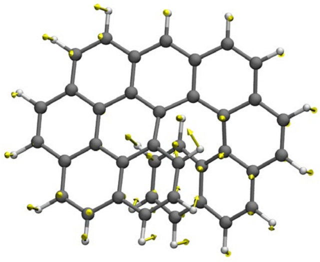

O9H-S_1-mode 10 (331)

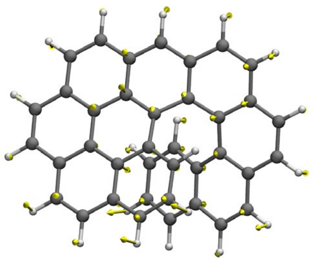

O9H-S_1-mode 15 (1466)

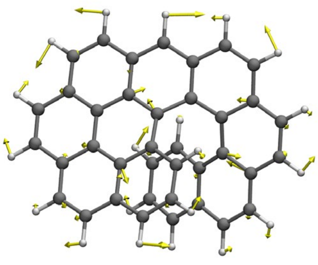

O9H-S_1-mode 122 (1629)

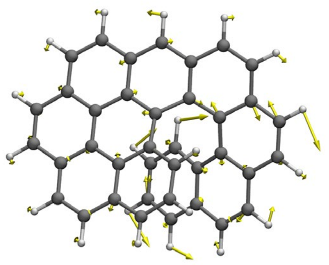

O9H-S_1-mode 136 (1668)

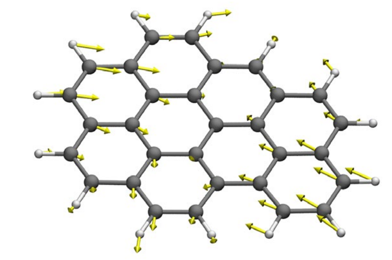

OO7H-S_0-mode 12 (323)

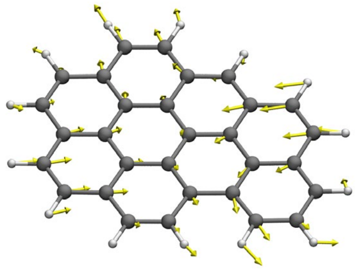

OO7H-S_0-mode 14 (334)

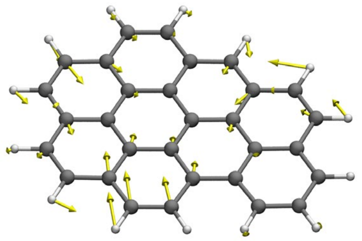

OO7H-S_0-mode 21 (463)

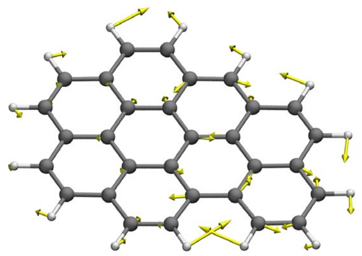

OO7H-S_0-mode 87 (1381)

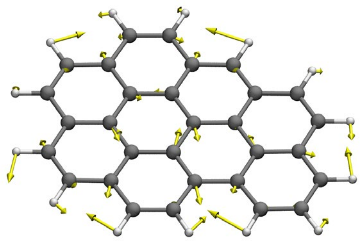

OO7H-S_0-mode 89 (1412)

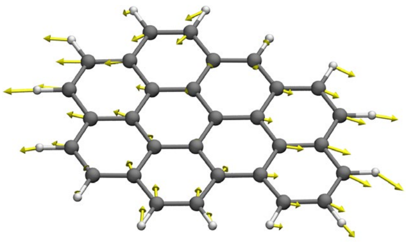

OO7H-S_1-mode 12 (312)

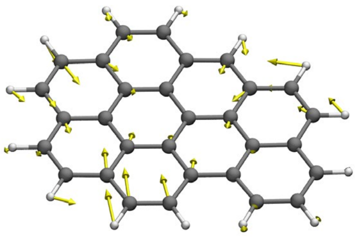

OO7H-S_1-mode 22 (459)

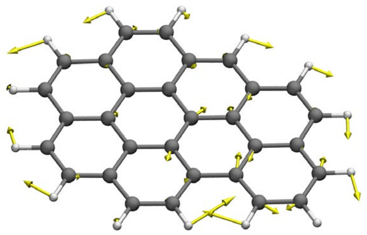

OO7H-S_1-mode 88 (1383)

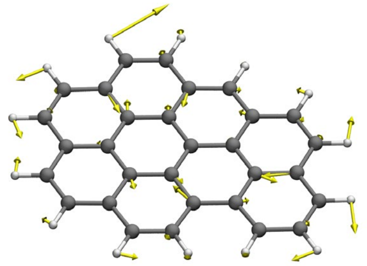

OO7H-S_1-mode 90 (1413)

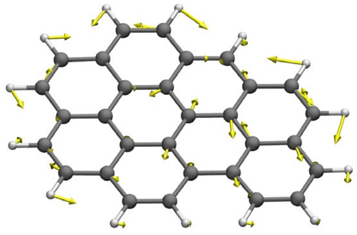

OO7H-S_1-mode 112 (1656)

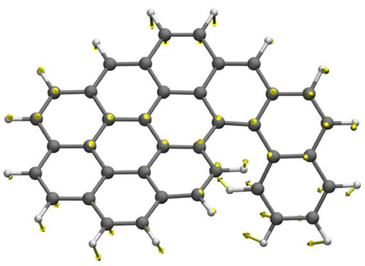

OO9H-S_0-mode 3 (116)

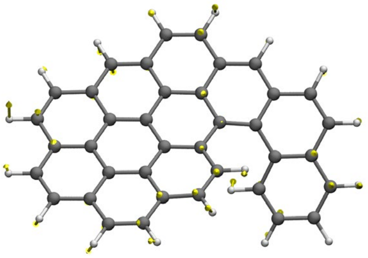

OO9H-S_0-mode 9 (327)

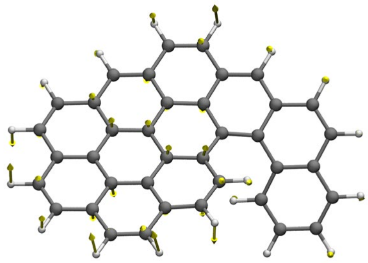

OO9H-S_0-mode 17 (367)

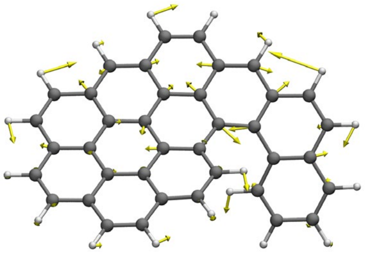

OO9H-S_0-mode 112 (1517)

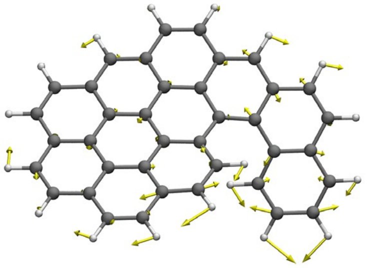

OO9H-S_0-mode 134 (1675)

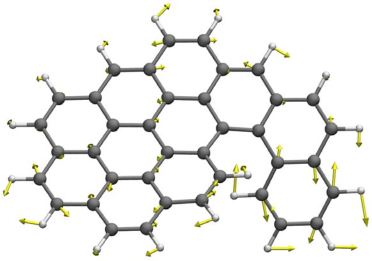

OO9H-S_0-mode 141 (1686)

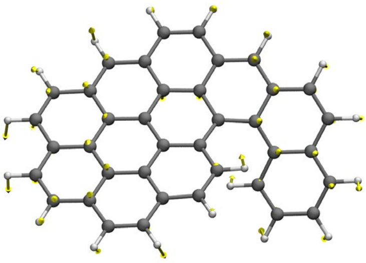

OO9H-S_1-mode 12 (117)

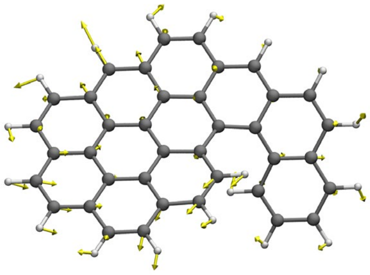

OO9H-S_1-mode 22 (271)

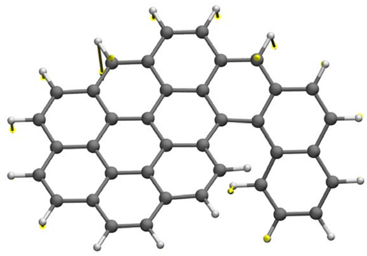

OO9H-S_1-mode 72 (366)

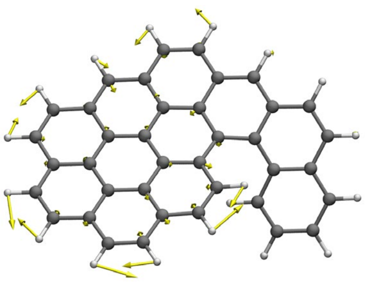

OO9H-S_1-mode 88 (1561)

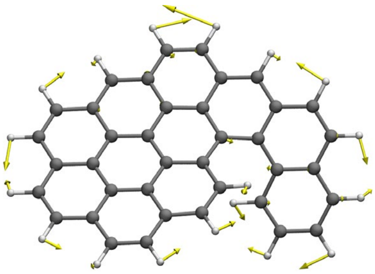

OO9H-S_1-mode 90 (1602)

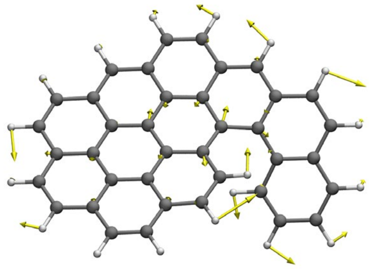

OO9H-S_1-mode 112 (1674)

5. Geometries of S_0 and S_1

Figure S6. Geometry comparison of ground states S_0 (in green), excited states S_1 (in blue).

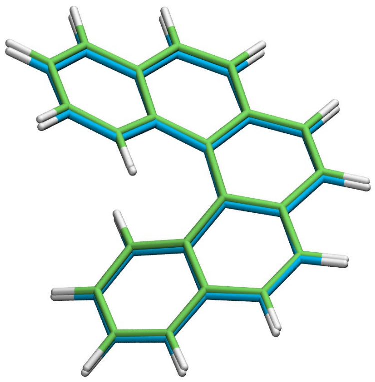

5H

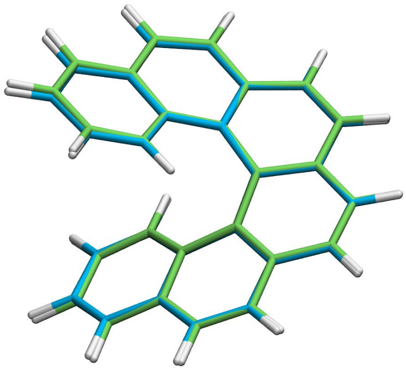

6H

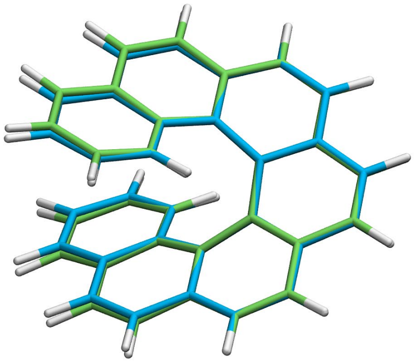

7H

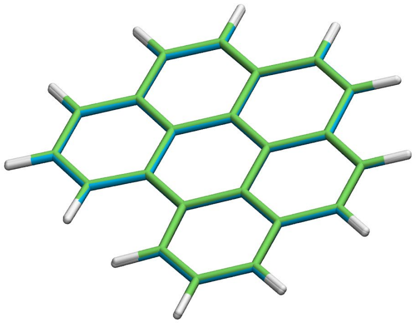

BP

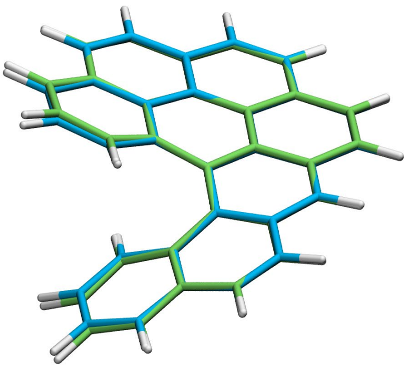

O7H

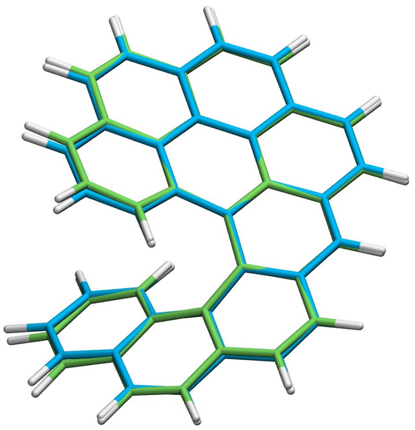

O8H

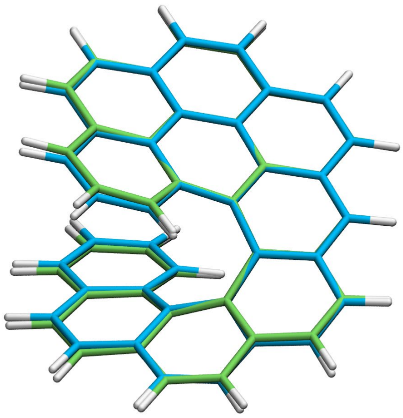

O9H

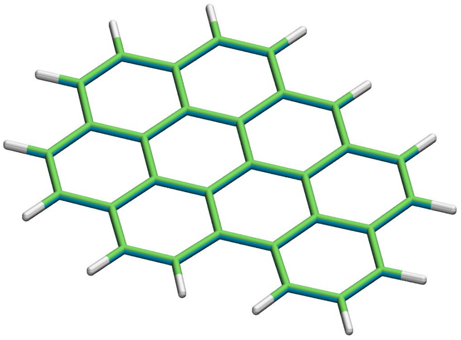

OO7H

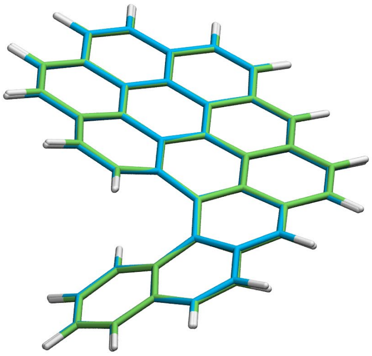

OO9H

6. Spectrum of OO7H and OO9H.

Figure S7. Pure calculated electronic spectra of **OO7H** and **OO9H** compared to experimental data.

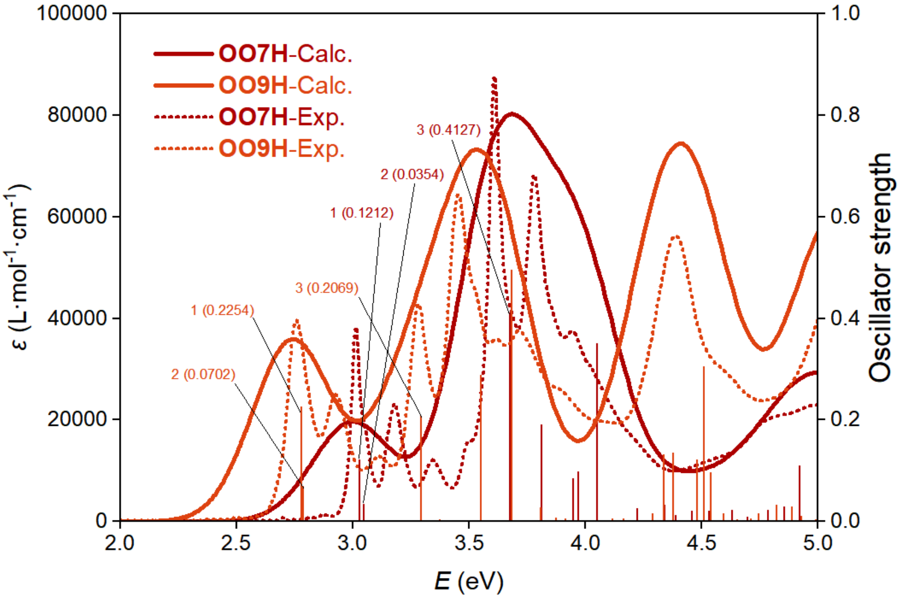

Figure S8. ABS spectrum of **OO7H** and **OO9H** using TD approach at 0 K.

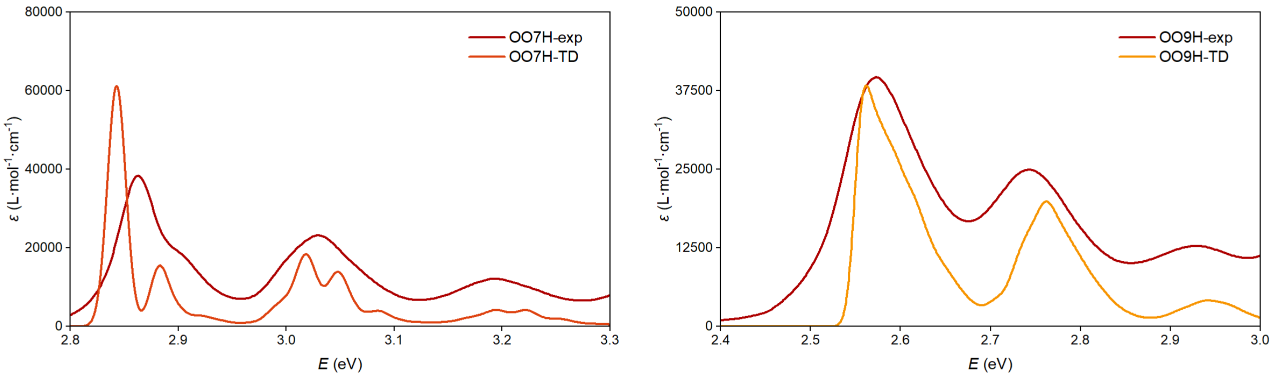

Figure S9. ABS and EMI spectrum of **OO7H** and **OO9H** using TI approach at 0 K.

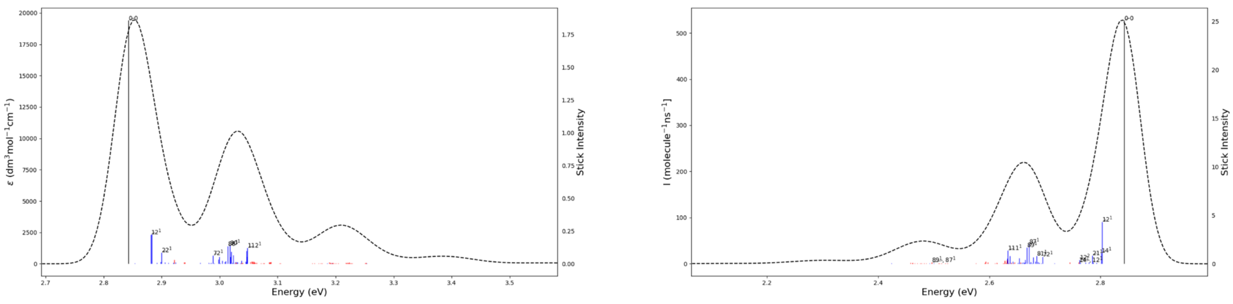

OO7H-ABS **OO7H-EMI**

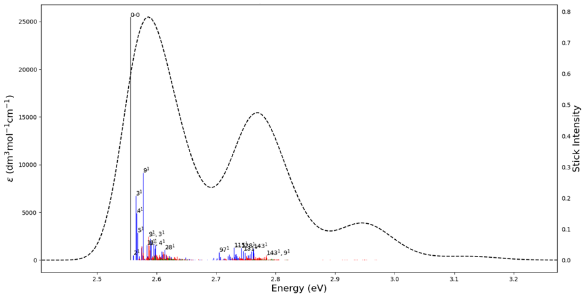

OO9H-ABS

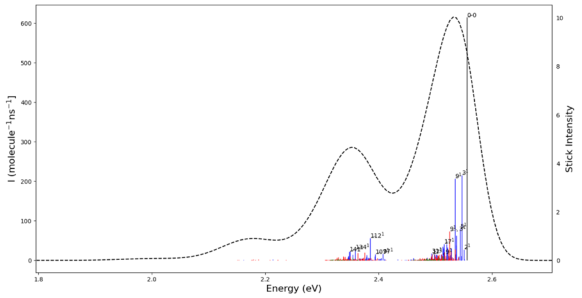

OO9H-EMI

7. Cartesian coordinates of the optimized geometries (in Å)

5H-S_0

C	-0.6479160	3.2336720	-0.2027340
C	0.6479210	3.2336710	0.2027350
C	-1.3615490	2.0158800	-0.3195040
C	1.3615520	2.0158780	0.3195050
C	-0.7171930	0.7886780	-0.0529110
C	0.7171940	0.7886780	0.0529150
C	2.7411960	2.0384970	0.6708080
C	1.5636100	-0.3611400	-0.1900380
C	3.4841430	0.9071310	0.6708640
C	2.9330100	-0.3087370	0.1800850
C	1.1286010	-1.4958980	-0.9058840
C	3.7571290	-1.4325600	-0.0285610
C	1.9606390	-2.5623460	-1.1358900
C	3.2803470	-2.5503140	-0.6620980
C	-2.7411920	2.0385000	-0.6708090
C	-1.5636100	-0.3611390	0.1900390
C	-3.4841400	0.9071340	-0.6708660
C	-2.9330100	-0.3087340	-0.1800860
H	-1.1751580	4.1642820	-0.3835710
H	1.1751640	4.1642810	0.3835690
H	3.1825980	2.9908800	0.9452480
H	4.5279680	0.9258690	0.9657080
H	0.1203830	-1.5199280	-1.2949150
H	4.7927550	-1.3804530	0.2913450
H	1.5952060	-3.4143020	-1.6979530
H	3.9275120	-3.4035860	-0.8303260
H	-3.1825920	2.9908830	-0.9452510
C	-1.1286040	-1.4958970	0.9058860
H	-4.5279640	0.9258730	-0.9657120
C	-3.7571300	-1.4325560	0.0285580
C	-3.2803510	-2.5503110	0.6620970
C	-1.9606440	-2.5623440	1.1358910
H	-0.1203870	-1.5199280	1.2949210
H	-4.7927560	-1.3804480	-0.2913490
H	-3.9275180	-3.4035820	0.8303230
H	-1.5952130	-3.4143000	1.6979550

5H-S_1

C	-0.6572750	3.2503340	-0.2134070
C	0.6572760	3.2503340	0.2134080
C	-1.3680430	2.0440790	-0.3421830
C	1.3680440	2.0440790	0.3421830

C	-0.7149970	0.7899400	-0.0346150
C	0.7149980	0.7899400	0.0346150
C	2.7189030	2.0291810	0.7341000
C	1.5471030	-0.3410410	-0.2421200
C	3.4651500	0.8761190	0.6952720
C	2.9266500	-0.3172600	0.1574700
C	1.1000420	-1.4715800	-0.9599190
C	3.7271140	-1.4493410	-0.0459120
C	1.9226960	-2.5583520	-1.1759750
C	3.2348170	-2.5673120	-0.6941400
C	-2.7189030	2.0291820	-0.7341010
C	-1.5471030	-0.3410400	0.2421210
C	-3.4651490	0.8761190	-0.6952740
C	-2.9266500	-0.3172590	-0.1574700
H	-1.1758950	4.1838620	-0.4011940
H	1.1758960	4.1838620	0.4011950
H	3.1726900	2.9599550	1.0583830
H	4.5034800	0.8854290	1.0090730
H	0.0960080	-1.4751540	-1.3619620
H	4.7581170	-1.4225460	0.2916260
H	1.5448720	-3.4093930	-1.7312640
H	3.8697750	-3.4307790	-0.8534380
H	-3.1726890	2.9599550	-1.0583840
C	-1.1000430	-1.4715790	0.9599200
H	-4.5034790	0.8854290	-1.0090750
C	-3.7271140	-1.4493400	0.0459110
C	-3.2348180	-2.5673110	0.6941400
C	-1.9226970	-2.5583510	1.1759760
H	-0.0960090	-1.4751540	1.3619630
H	-4.7581170	-1.4225450	-0.2916280
H	-3.8697770	-3.4307780	0.8534380
H	-1.5448740	-3.4093920	1.7312650

6H-S$_0$

C	1.1640630	3.5682230	0.3902900
C	2.2895100	2.8999140	0.7493110
C	0.0000300	2.8599900	0.0000030
C	2.3849520	1.5010730	0.5411940
C	0.0000160	1.4431890	0.0000050
C	1.2811770	0.7836820	0.0364790
C	3.6147760	0.8308750	0.8003690
C	1.5355650	-0.5304720	-0.5118700
C	3.7801710	-0.4752630	0.4864380
C	2.7672380	-1.1747060	-0.2274850

C	0.6478570	-1.1813450	-1.3911350
C	3.0041650	-2.4748120	-0.7146040
C	0.9146290	-2.4344180	-1.8819370
C	2.0888680	-3.1062080	-1.5162800
C	-1.1639900	3.5682450	-0.3902860
C	-1.2811580	0.7837060	-0.0364740
C	-2.2894470	2.8999570	-0.7493120
C	-2.3849160	1.5011180	-0.5411960
H	1.1202590	4.6512170	0.4343060
H	3.1587240	3.4310590	1.1221440
H	4.4183660	1.3967220	1.2599840
H	4.7098590	-0.9880560	0.7093840
H	-0.2567590	-0.6763750	-1.6989780
H	3.9431760	-2.9578390	-0.4643480
H	0.2116880	-2.9032850	-2.5609810
H	2.2852830	-4.1038710	-1.8922110
H	-1.1201640	4.6512390	-0.4343050
C	-1.5355750	-0.5304430	0.5118730
H	-3.1586490	3.4311190	-1.1221500
C	-3.6147510	0.8309430	-0.8003810
C	-3.7801730	-0.4751920	-0.4864550
C	-2.7672580	-1.1746540	0.2274770
C	-0.6478910	-1.1813320	1.3911520
H	-4.4183260	1.3968060	-1.2600020
H	-4.7098680	-0.9879680	-0.7094080
C	-3.0042150	-2.4747560	0.7145940
C	-2.0889390	-3.1061680	1.5162800
C	-0.9146930	-2.4343990	1.8819520
H	0.2567290	-0.6763770	1.6990070
H	-3.9432320	-2.9577650	0.4643290
H	-2.2853770	-4.1038270	1.8922100
H	-0.2117690	-2.9032780	2.5610060

6H-S$_1$

C	1.1587550	3.5876970	0.4022850
C	2.3076460	2.9023860	0.7311740
C	0.0000480	2.9034510	0.0000040
C	2.4044050	1.5131510	0.5079970
C	0.0000250	1.4496880	0.0000060
C	1.2721510	0.7992960	-0.0180250
C	3.5976810	0.8167520	0.7761040
C	1.5080560	-0.5080120	-0.5709960
C	3.7410540	-0.5091730	0.4533990
C	2.7273530	-1.1890590	-0.2615700

C	0.6038340	-1.1488120	-1.4377270
C	2.9153710	-2.5075840	-0.7134330
C	0.8269780	-2.4284720	-1.8922320
C	1.9764250	-3.1289530	-1.5040470
C	-1.1586360	3.5877330	-0.4022790
C	-1.2721210	0.7993350	0.0180310
C	-2.3075460	2.9024580	-0.7311750
C	-2.4043490	1.5132260	-0.5080000
H	1.1241400	4.6693650	0.4758670
H	3.1778750	3.4322760	1.1025460
H	4.4133860	1.3553830	1.2471630
H	4.6601280	-1.0357740	0.6864940
H	-0.2835430	-0.6183830	-1.7563370
H	3.8321070	-3.0215330	-0.4430730
H	0.1057280	-2.8948230	-2.5533420
H	2.1376650	-4.1438570	-1.8487320
H	-1.1239860	4.6694000	-0.4758630
C	-1.5080730	-0.5079660	0.5710000
H	-3.1777560	3.4323740	-1.1025530
C	-3.5976440	0.8168640	-0.7761200
C	-3.7410610	-0.5090580	-0.4534190
C	-2.7273880	-1.1889760	0.2615600
C	-0.6038850	-1.1487920	1.4377470
H	-4.4133270	1.3555200	-1.2471860
H	-4.6601480	-1.0356300	-0.6865240
C	-2.9154520	-2.5074940	0.7134210
C	-1.9765370	-3.1288910	1.5040480
C	-0.8270750	-2.4284450	1.8922510
H	0.2835010	-0.6183890	1.7563740
H	-3.8322010	-3.0214150	0.4430490
H	-2.1378140	-4.1437900	1.8487330
H	-0.1058510	-2.8948170	2.5533740

7H-S_0

C	-2.8493550	2.7745390	0.4965550
C	-1.6845400	3.4709600	0.5376970
C	-2.8472390	1.3858290	0.2059320
C	-0.4744450	2.8718180	0.1016510
C	-1.6225700	0.7211110	-0.0334530
C	-0.4687550	1.5299170	-0.3248130
C	0.7324470	3.6272210	0.0858480
C	0.6811800	1.0654770	-1.0635700
C	1.8842340	3.0950600	-0.3880080
C	1.8794180	1.8217690	-1.0225770

C	0.6510480	-0.0685640	-1.8987330
C	3.0302490	1.3377720	-1.6741700
C	1.7758530	-0.5033290	-2.5532590
C	2.9899910	0.1827710	-2.4113480
C	-4.0662000	0.6616480	0.1505940
C	-1.6225240	-0.7212040	0.0334600
C	-4.0661590	-0.6618970	-0.1505790
C	-2.8471510	-1.3860000	-0.2059240
H	-3.7947710	3.2546770	0.7255790
H	-1.6696490	4.5132610	0.8381770
H	0.7104400	4.6366140	0.4833550
H	2.8108470	3.6584780	-0.3576740
H	-0.2808440	-0.5973780	-2.0395500
H	3.9466850	1.9145410	-1.6033460
H	1.7199530	-1.3798650	-3.1885550
H	3.8806070	-0.1778010	-2.9134270
H	-4.9963220	1.2007860	0.2953320
C	-0.4686560	-1.5299390	0.3248090
H	-4.9962470	-1.2010930	-0.2953140
C	-2.8491790	-2.7747070	-0.4965590
C	-1.6843180	-3.4710510	-0.5377180
C	-0.4742590	-2.8718340	-0.1016750
C	0.6812490	-1.0654360	1.0635730
H	-3.7945640	-3.2549050	-0.7255830
H	-1.6693610	-4.5133470	-0.8382100
C	0.7326840	-3.6271550	-0.0858940
C	1.8844390	-3.0949230	0.3879640
C	1.8795380	-1.8216470	1.0225620
C	0.6510400	0.0685790	1.8987690
H	0.7107440	-4.6365420	-0.4834170
H	2.8110900	-3.6582770	0.3576130
C	3.0303360	-1.3375900	1.6741700
C	1.7758140	0.5033990	2.5533110
C	2.9899980	-0.1826150	2.4113820
H	-0.2808890	0.5973270	2.0395980
H	3.9468110	-1.9142950	1.6033310
H	1.7198540	1.3799130	3.1886330
H	3.8805880	0.1780010	2.9134760

7H-S_1

C	-2.8602030	2.8004510	0.3407250
C	-1.6701440	3.4922070	0.3614460
C	-2.8821430	1.4129430	0.1133070
C	-0.4694510	2.8622540	-0.0207910

C	-1.6392090	0.7090730	-0.0892290
C	-0.4859780	1.4891230	-0.4229600
C	0.7643440	3.5500390	0.0073620
C	0.6601500	0.9732330	-1.1172860
C	1.9196290	2.9556940	-0.4157450
C	1.8955140	1.6779320	-1.0327100
C	0.6228200	-0.1975070	-1.8978580
C	3.0524480	1.1141790	-1.5914320
C	1.7679290	-0.7157220	-2.4619260
C	2.9987270	-0.0776740	-2.2812600
C	-4.0838900	0.6828330	0.0925500
C	-1.6392100	-0.7090710	0.0892280
C	-4.0838910	-0.6828280	-0.0925510
C	-2.8821450	-1.4129400	-0.1133070
H	-3.7964950	3.3081930	0.5461170
H	-1.6469140	4.5431030	0.6280390
H	0.7802990	4.5636420	0.3946850
H	2.8653670	3.4828450	-0.3478910
H	-0.3258340	-0.6898100	-2.0644800
H	3.9925490	1.6455880	-1.4855960
H	1.7091960	-1.6241260	-3.0503040
H	3.8997740	-0.5017260	-2.7091880
H	-5.0211050	1.2212940	0.1857480
C	-0.4859790	-1.4891230	0.4229590
H	-5.0211060	-1.2212880	-0.1857500
C	-2.8602070	-2.8004480	-0.3407240
C	-1.6701480	-3.4922060	-0.3614440
C	-0.4694540	-2.8622540	0.0207920
C	0.6601500	-0.9732330	1.1172850
H	-3.7964990	-3.3081890	-0.5461150
H	-1.6469190	-4.5431030	-0.6280350
C	0.7643390	-3.5500400	-0.0073610
C	1.9196250	-2.9556970	0.4157450
C	1.8955120	-1.6779340	1.0327100
C	0.6228200	0.1975070	1.8978570
H	0.7802940	-4.5636430	-0.3946840
H	2.8653630	-3.4828480	0.3478910
C	3.0524470	-1.1141820	1.5914320
C	1.7679300	0.7157210	2.4619250
C	2.9987280	0.0776710	2.2812590
H	-0.3258330	0.6898100	2.0644800
H	3.9925470	-1.6455920	1.4855970
H	1.7091980	1.6241240	3.0503040
H	3.8997750	0.5017220	2.7091870

BP-S_0

C	1.4225310	-0.3423290	0.0076010
C	2.8422150	-0.3214410	0.0016260
C	3.5198710	0.9339700	0.0033390
C	2.8336660	2.1011380	0.0037220
C	0.7303760	-1.5807470	-0.0049400
C	3.5472190	-1.5293520	-0.0013100
H	4.6048010	0.9319520	0.0034900
H	3.3597580	3.0500950	0.0054990
C	2.8684780	-2.7288320	-0.0058100
C	1.4790670	-2.7535140	-0.0067480
H	4.6318310	-1.5086370	-0.0021300
H	3.4178990	-3.6634380	-0.0089050
H	0.9825810	-3.7149870	-0.0123540
C	0.6835010	3.3231760	0.0012740
H	1.2308100	4.2600280	0.0028140
C	-0.6834430	3.3231810	-0.0012650
H	-1.2307350	4.2600430	-0.0028000
C	-1.4091570	2.1147730	-0.0015980
C	-0.7093520	0.8905260	0.0050360
C	-2.8336370	2.1011720	-0.0037260
C	-1.4225340	-0.3423130	-0.0076030
C	-3.5198650	0.9340140	-0.0033450
H	-3.3596950	3.0501480	-0.0055060
C	-0.7304010	-1.5807360	0.0049400
C	-2.8422220	-0.3214090	-0.0016300
H	-4.6047940	0.9319900	-0.0035040
C	-1.4791120	-2.7534960	0.0067520
C	-3.5472450	-1.5293040	0.0013070
C	-2.8685200	-2.7287960	0.0058130
H	-0.9826340	-3.7149730	0.0123600
H	-4.6318580	-1.5085830	0.0021240
H	-3.4179620	-3.6633900	0.0089120
C	1.4091940	2.1147560	0.0016000
C	0.7093690	0.8905210	-0.0050360

BP-S_1

C	1.4124830	-0.3383310	0.0081660
C	2.8372250	-0.3215410	0.0025640
C	3.5092410	0.9156620	0.0076600
C	2.8140700	2.1147930	0.0093740
C	0.7159130	-1.5675510	-0.0097660
C	3.5338480	-1.5482100	-0.0026520

H	4.5939310	0.9189070	0.0091080
H	3.3555680	3.0547220	0.0136890
C	2.8456900	-2.7507770	-0.0136050
C	1.4646120	-2.7768630	-0.0134700
H	4.6184520	-1.5366650	-0.0021880
H	3.3976750	-3.6840760	-0.0213470
H	0.9556460	-3.7300320	-0.0370540
C	0.6779730	3.3560030	0.0027380
H	1.2276460	4.2913500	0.0053110
C	-0.6779740	3.3560030	-0.0027430
H	-1.2276470	4.2913490	-0.0053190
C	-1.4198610	2.1359350	-0.0050780
C	-0.7021890	0.8951830	0.0033600
C	-2.8140710	2.1147930	-0.0093720
C	-1.4124830	-0.3383310	-0.0081650
C	-3.5092410	0.9156620	-0.0076560
H	-3.3555690	3.0547210	-0.0136860
C	-0.7159120	-1.5675510	0.0097660
C	-2.8372250	-0.3215420	-0.0025630
H	-4.5939310	0.9189050	-0.0091020
C	-1.4646120	-2.7768640	0.0134690
C	-3.5338470	-1.5482100	0.0026530
C	-2.8456890	-2.7507780	0.0136030
H	-0.9556440	-3.7300320	0.0370520
H	-4.6184510	-1.5366670	0.0021890
H	-3.3976750	-3.6840760	0.0213450
C	1.4198610	2.1359350	0.0050770
C	0.7021880	0.8951830	-0.0033600

O7H-S_0

C	0.3279950	2.7208220	-0.2060720
C	-0.9916530	3.0508490	-0.4602000
C	0.6830180	1.3427070	-0.1235400
C	-1.9952860	2.0845160	-0.4202450
C	-0.3067370	0.3390110	-0.2216490
C	-1.6730990	0.7253420	-0.1502180
C	-3.3580430	2.4613290	-0.6423580
C	-2.7677870	-0.1397320	0.2600310
C	-4.3632680	1.5734210	-0.4984520
C	-4.1033850	0.2693360	0.0238990
C	-2.5724580	-1.3116410	1.0151580
C	-5.1681310	-0.5700220	0.3985470
C	-3.6280690	-2.0984240	1.4077200
C	-4.9398100	-1.7467840	1.0671210

C	2.0515910	0.9992070	0.0933390
C	0.1494010	-1.0258400	-0.4703660
C	2.4451770	-0.3604800	0.1305490
C	1.5005000	-1.3673170	-0.2053520
C	1.3409400	3.7176960	-0.0580400
H	-1.2628360	4.0868350	-0.6377660
H	-3.5593130	3.4856050	-0.9379580
H	-5.3915220	1.8599790	-0.6921220
H	-1.5691220	-1.5945450	1.3014340
H	-6.1816120	-0.2491700	0.1805710
H	-3.4408590	-2.9906660	1.9943390
H	-5.7689550	-2.3793690	1.3628830
C	3.0151610	2.0080440	0.2984070
C	-0.6605600	-1.9945130	-1.0609290
C	3.7893780	-0.7020740	0.3911540
C	1.9414710	-2.7087230	-0.3543840
C	1.0557940	-3.6672010	-0.8544350
C	-0.2155930	-3.2961540	-1.2452260
H	-1.6624850	-1.7324220	-1.3711130
C	3.2930860	-3.0322600	-0.0269950
H	1.3934070	-4.6914380	-0.9721150
H	-0.8794110	-4.0248930	-1.6963390
C	4.1753510	-2.0735590	0.3433540
C	2.6231910	3.3776500	0.2031650
C	4.3431270	1.6407780	0.5874210
C	4.7186950	0.3247010	0.6420400
H	1.0498200	4.7596740	-0.1378280
H	3.6058480	-4.0683220	-0.1039880
H	5.2041370	-2.3303190	0.5732540
H	3.3803180	4.1424300	0.3409240
H	5.0735940	2.4239440	0.7615740
H	5.7490090	0.0585750	0.8535790

O7H-S_1

C	0.3783250	2.7288970	-0.1650520
C	-0.9539040	3.0757260	-0.4699090
C	0.7157650	1.3476660	-0.0642850
C	-1.9737770	2.1358400	-0.4571320
C	-0.2862770	0.3610920	-0.1985910
C	-1.6786010	0.7758100	-0.1480980
C	-3.3396870	2.5152720	-0.6611760
C	-2.7271780	-0.0622250	0.2889290
C	-4.3477180	1.6380020	-0.4525570
C	-4.0867330	0.3376570	0.0710190

C	-2.5006810	-1.2928020	0.9830840
C	-5.1222780	-0.5371900	0.4005230
C	-3.5532220	-2.1156250	1.3179070
C	-4.8641950	-1.7617470	0.9957820
C	2.0732300	0.9779130	0.1073260
C	0.1095250	-0.9818330	-0.4580260
C	2.4349140	-0.3863240	0.0906580
C	1.4606760	-1.3620770	-0.2388800
C	1.3921010	3.6910870	-0.0097490
H	-1.1979670	4.1182400	-0.6494060
H	-3.5463920	3.5303670	-0.9838940
H	-5.3783300	1.9260730	-0.6319150
H	-1.5069210	-1.5230830	1.3387130
H	-6.1448560	-0.2318820	0.2038850
H	-3.3590950	-3.0355090	1.8580520
H	-5.6841320	-2.4214470	1.2556020
C	3.0689640	1.9763650	0.3108070
C	-0.7887390	-1.9627250	-0.9683110
C	3.7855160	-0.7680320	0.3138540
C	1.8495290	-2.7236290	-0.3614220
C	0.8918930	-3.6767680	-0.7734200
C	-0.3872800	-3.2844620	-1.1069670
H	-1.7578330	-1.6551800	-1.3324170
C	3.1881580	-3.0773070	-0.0883910
H	1.1929360	-4.7143460	-0.8711870
H	-1.0913080	-4.0118850	-1.4954370
C	4.1264770	-2.1287690	0.2422250
C	2.6976180	3.3271150	0.2487310
C	4.4082340	1.5628290	0.5568970
C	4.7498580	0.2467830	0.5704830
H	1.1302340	4.7403130	-0.0972680
H	3.4730650	-4.1218720	-0.1572300
H	5.1540090	-2.4226140	0.4292130
H	3.4567550	4.0888230	0.3902370
H	5.1599940	2.3256070	0.7305080
H	5.7767460	-0.0508310	0.7549810

O8H-S$_0$

C	-2.2889650	3.5473260	-0.5962140
C	-3.4529370	2.8795910	-0.7609260
C	-1.0837580	2.8447810	-0.3013630
C	-3.5414230	1.4948230	-0.4374200
C	-1.1040060	1.4206620	-0.2143480
C	-2.3943610	0.7905340	-0.0316460

C	-4.8054140	0.8444700	-0.4696180
C	-2.5886370	-0.4696350	0.6440210
C	-4.9484520	-0.4308310	-0.0334940
C	-3.8580250	-1.1021790	0.5826410
C	-1.5918740	-1.0825340	1.4301050
C	-4.0404620	-2.3613030	1.1887560
C	-1.8025280	-2.2931430	2.0383230
C	-3.0277950	-2.9595320	1.8915610
C	0.1119530	3.5416000	-0.1278680
C	0.1444460	0.7431050	-0.3221640
C	1.2992950	2.8754270	0.1175350
C	1.3272860	1.4577090	-0.0367750
H	-2.2380860	4.6232180	-0.7245120
H	-4.3579800	3.4011390	-1.0535830
H	-5.6560530	1.3962860	-0.8557860
H	-5.9088370	-0.9326600	-0.0862120
H	-0.6461990	-0.5782820	1.5710040
H	-5.0122520	-2.8376790	1.1088970
H	-1.0157710	-2.7330060	2.6404740
H	-3.1813710	-3.9252710	2.3595130
H	0.0979360	4.6266660	-0.1549730
C	0.2847960	-0.6293250	-0.7956580
C	2.5013750	3.5676480	0.4603900
C	2.5684030	0.7726390	0.1345230
C	2.6386480	-0.6271740	-0.0657460
C	1.5054600	-1.3161210	-0.5767350
C	-0.7146510	-1.2869400	-1.5084410
C	3.7227740	1.4792820	0.5282300
C	3.8530410	-1.3129410	0.1507190
C	1.6263070	-2.6864450	-0.9260200
C	0.5573590	-3.3266960	-1.5597590
C	-0.5810930	-2.6167870	-1.8824540
H	-1.6195230	-0.7558200	-1.7689300
C	2.8514050	-3.3646460	-0.6486250
H	0.6493040	-4.3743460	-1.8259590
H	-1.3871410	-3.0990240	-2.4234430
C	3.6561850	2.8981220	0.6761690
C	4.9153250	0.7687400	0.7633080
C	4.9777050	-0.5883210	0.5885540
C	3.9146910	-2.7104820	-0.1223260
H	2.4600270	4.6468470	0.5626990
H	2.9187500	-4.4207240	-0.8882870
H	4.5578560	3.4304450	0.9603780
H	5.7946880	1.3184360	1.0824350

H	5.9073850	-1.1196440	0.7633220
H	4.8448220	-3.2351190	0.0695580

O8H-S$_1$

C	-2.2883100	3.6057520	-0.5627670
C	-3.4764800	2.9508380	-0.6075740
C	-1.0729490	2.8939370	-0.3340790
C	-3.5563740	1.5723690	-0.2916200
C	-1.1179370	1.4668820	-0.2019230
C	-2.3622830	0.8593610	0.0580270
C	-4.7794840	0.8835300	-0.3616720
C	-2.5232110	-0.4606280	0.6596360
C	-4.8719290	-0.4459180	-0.0405550
C	-3.7674540	-1.1333380	0.5166060
C	-1.5406400	-1.0606250	1.4605470
C	-3.8995630	-2.4472240	1.0150520
C	-1.7069640	-2.3261530	1.9774890
C	-2.8832870	-3.0411690	1.7202480
C	0.1330080	3.5592650	-0.1731960
C	0.1483420	0.7627530	-0.3237150
C	1.3340200	2.8746040	0.1035680
C	1.3434270	1.4564720	-0.0210460
H	-2.2348620	4.6757430	-0.7329300
H	-4.3911630	3.4789120	-0.8561050
H	-5.6582780	1.4275570	-0.6921430
H	-5.8186450	-0.9678240	-0.1309440
H	-0.6271320	-0.5185240	1.6676260
H	-4.8400200	-2.9666710	0.8620480
H	-0.9210570	-2.7681820	2.5786660
H	-3.0022570	-4.0477000	2.1053380
H	0.1411170	4.6446480	-0.1998410
C	0.2461520	-0.5807550	-0.7963020
C	2.5285300	3.5411230	0.4268660
C	2.5682360	0.7594940	0.1196380
C	2.6113940	-0.6340890	-0.1069900
C	1.4575660	-1.2965840	-0.5965790
C	-0.8222190	-1.2498360	-1.4374930
C	3.7536530	1.4564610	0.4924860
C	3.8192120	-1.3500050	0.0924220
C	1.5216720	-2.6847490	-0.8884300
C	0.3892480	-3.3259540	-1.4284190
C	-0.7439420	-2.5989030	-1.7370800
H	-1.7036270	-0.6904380	-1.7167210
C	2.7280270	-3.3804430	-0.6392590

H	0.4374660	-4.3885640	-1.6410360
H	-1.5885920	-3.0867690	-2.2098850
C	3.7031730	2.8495800	0.6429280
C	4.9477130	0.7091870	0.6969690
C	4.9766610	-0.6380680	0.5166290
C	3.8399560	-2.7335580	-0.1650560
H	2.5153220	4.6232300	0.5047680
H	2.7632690	-4.4454670	-0.8430750
H	4.6069210	3.3834010	0.9161590
H	5.8437230	1.2409690	0.9993550
H	5.8955460	-1.1925950	0.6754900
H	4.7604840	-3.2833640	0.0011020

O9H-S_0

C	1.3455340	3.0884210	0.1009250
C	0.1577350	3.7954530	0.0212620
C	1.3333100	1.6964170	-0.2135640
C	-1.0622880	3.1352150	-0.1395300
C	0.1233700	1.0428720	-0.5172640
C	-1.0956610	1.7146370	-0.2321760
C	-2.2828090	3.8681660	-0.2422940
C	-2.3730220	1.0603780	-0.0287210
C	-3.4586780	3.2245440	-0.4229420
C	-3.5377500	1.8051850	-0.3060670
C	-2.5306610	-0.2880970	0.4347850
C	-4.7802080	1.1408210	-0.4474950
C	-3.7405780	-0.9505080	0.1463890
C	-4.8564790	-0.2108940	-0.3178270
C	2.5536260	0.9592020	-0.1442980
C	0.1978410	-0.2836830	-1.1119650
C	2.5630440	-0.4239010	-0.4474440
C	1.3929900	-1.0307650	-0.9774250
C	2.5838610	3.7125660	0.4470680
H	0.1641290	4.8759400	0.1253500
H	-2.2357230	4.9517500	-0.2260330
H	-4.3761090	3.7787890	-0.5904940
C	-1.5332390	-1.0088350	1.1926010
H	-5.6571070	1.7262000	-0.7028980
C	-3.8339480	-2.3620370	0.3168610
H	-5.7864020	-0.7360530	-0.5084820
C	3.7415370	1.5911230	0.2741160
C	-0.8396200	-0.8354330	-1.8593430
C	3.7514440	-1.1706520	-0.3069580
C	1.4468940	-2.3755740	-1.4244390

C	0.3391140	-2.9204340	-2.0801400
C	-0.7708080	-2.1389050	-2.3322210
H	-1.7208980	-0.2438660	-2.0655300
C	2.6464380	-3.1217300	-1.2180770
H	0.3781850	-3.9496390	-2.4210210
H	-1.6050720	-2.5448010	-2.8926750
C	3.7465020	-2.5492090	-0.6721910
C	3.7257840	2.9938590	0.5452540
C	4.9110010	0.8209990	0.4226960
C	4.9143640	-0.5219710	0.1503250
H	2.5812780	4.7770110	0.6566150
H	2.6639950	-4.1615790	-1.5275550
H	4.6564520	-3.1242130	-0.5354230
H	4.6543170	3.4730780	0.8376700
H	5.8187760	1.3111760	0.7589720
H	5.8259020	-1.0995140	0.2637400
C	-1.6289030	-2.4191220	1.2896670
C	-2.7892030	-3.0789800	0.7944720
C	-0.5018270	-0.3717920	1.9096820
C	-0.6083330	-3.1418550	1.9368800
C	0.4650950	-1.0945640	2.5626570
C	0.4385370	-2.4956450	2.5422490
H	-4.7569180	-2.8532480	0.0266650
H	-2.8471730	-4.1593610	0.8746470
H	-0.4753010	0.7077490	1.9531670
H	-0.6790600	-4.2242510	1.9673890
H	1.2517850	-0.5753690	3.0980140
H	1.2209080	-3.0614450	3.0348330

O9H-S_1

C	1.3211230	3.0935200	0.0674910
C	0.1080800	3.8067510	-0.0520950
C	1.3160310	1.6965550	-0.2033680
C	-1.1076880	3.1619740	-0.2062800
C	0.1069880	1.0402320	-0.5165690
C	-1.1445660	1.7294240	-0.2503040
C	-2.3461060	3.8773240	-0.2411400
C	-2.3559990	1.0728180	0.0214810
C	-3.5266870	3.2153910	-0.2843630
C	-3.5773290	1.7966090	-0.1680750
C	-2.4542030	-0.3342000	0.4002470
C	-4.7704690	1.0871850	-0.3283690
C	-3.6547310	-1.0238690	0.0876030
C	-4.7892540	-0.2959640	-0.2983020

C	2.5322950	0.9716600	-0.1670260
C	0.1586540	-0.2515380	-1.1088550
C	2.5338090	-0.4047820	-0.4870470
C	1.3542880	-1.0065580	-0.9916150
C	2.5410730	3.7150740	0.3960060
H	0.1227920	4.8880580	0.0450120
H	-2.3151150	4.9615370	-0.2635000
H	-4.4610780	3.7569120	-0.3903810
C	-1.4708740	-1.0042520	1.1875430
H	-5.6825250	1.6394570	-0.5288600
C	-3.6898590	-2.4435220	0.2272530
H	-5.7089810	-0.8329190	-0.5043970
C	3.7355560	1.6144420	0.2262270
C	-0.9507980	-0.8419940	-1.7578380
C	3.7252570	-1.1608020	-0.3595230
C	1.3670810	-2.3759400	-1.3580620
C	0.2020230	-2.9485150	-1.9107640
C	-0.9136570	-2.1746130	-2.1483920
H	-1.8018650	-0.2282790	-2.0156760
C	2.5531140	-3.1199850	-1.1617280
H	0.2131660	-3.9974450	-2.1872330
H	-1.7806190	-2.6082070	-2.6330970
C	3.6953820	-2.5316430	-0.6831500
C	3.7100600	2.9953980	0.4951800
C	4.9166770	0.8321540	0.3417140
C	4.9080050	-0.5032260	0.0744070
H	2.5463440	4.7839620	0.5826910
H	2.5505600	-4.1751350	-1.4146670
H	4.6004850	-3.1179330	-0.5631400
H	4.6318090	3.4919550	0.7788880
H	5.8332860	1.3214410	0.6541780
H	5.8178460	-1.0861000	0.1735120
C	-1.5117320	-2.4240770	1.2767670
C	-2.6356520	-3.1201680	0.7416440
C	-0.4363660	-0.3252510	1.8696360
C	-0.4617120	-3.1044890	1.9068480
C	0.5726170	-1.0196570	2.4963770
C	0.5841410	-2.4175520	2.4858400
H	-4.5835890	-2.9695440	-0.0923850
H	-2.6538040	-4.2029490	0.8106690
H	-0.4621920	0.7539430	1.9278810
H	-0.4890590	-4.1889140	1.9379160
H	1.3609780	-0.4755780	3.0041570
H	1.3941360	-2.9577730	2.9620180

OO7H-S_0

C	0.5078630	2.7624770	-0.0002300
C	-0.8162540	3.2011180	0.0020770
C	0.7756830	1.3675940	-0.0042710
C	-1.8783880	2.3085850	0.0010610
C	-0.2910050	0.4436090	0.0069280
C	-1.6204890	0.9081920	-0.0035140
C	-3.2369000	2.7594140	0.0016360
C	-2.6998320	-0.0176860	0.0059430
C	-4.2628140	1.8822910	0.0007830
C	-4.0354580	0.4683390	0.0002190
C	-2.4529430	-1.4150160	-0.0032600
C	-5.0943350	-0.4362890	-0.0009110
C	-3.5516480	-2.2800250	-0.0028230
C	-4.8489950	-1.7987940	-0.0026720
C	2.1213380	0.9103280	0.0048790
C	-0.0261700	-0.9621510	-0.0074250
C	2.3924780	-0.4806440	-0.0061040
C	1.3210870	-1.4140410	0.0048500
C	1.6050190	3.6720910	-0.0000010
H	-1.0202100	4.2675420	0.0046900
H	-3.4233920	3.8282150	0.0021130
H	-5.2890910	2.2343250	0.0002550
H	-6.1121580	-0.0610170	-0.0023200
H	-3.3994100	-3.3511040	-0.0052950
H	-5.6778400	-2.4976040	-0.0035660
C	3.1851960	1.8360250	-0.0008110
C	-1.0839340	-1.8909710	0.0020300
C	3.7298170	-0.9359120	-0.0009620
C	1.6054600	-2.7974190	0.0010930
C	0.5295000	-3.7046420	0.0016800
C	-0.7652960	-3.2652490	0.0023510
C	2.9581910	-3.2290050	0.0007020
H	0.7408780	-4.7688280	0.0014530
H	-1.5591420	-4.0003210	0.0040550
C	3.9811350	-2.3331720	0.0002060
C	2.8872300	3.2277760	-0.0011360
C	4.5117570	1.3542050	-0.0015980
C	4.7756200	0.0129490	-0.0011880
H	1.3922400	4.7359610	0.0002330
H	3.1612370	-4.2948230	0.0010470
H	5.0107930	-2.6752630	0.0007070
H	3.7117910	3.9330760	-0.0019960

H	5.3264530	2.0708490	-0.0028520
H	5.8008470	-0.3421510	-0.0005640

OO7H-S_1

C	0.5397210	2.7615360	0.0003960
C	-0.7812830	3.2050330	0.0022330
C	0.8010660	1.3605340	-0.0060260
C	-1.8714210	2.3140400	0.0008740
C	-0.2813910	0.4398520	0.0084840
C	-1.6264310	0.9156920	-0.0058090
C	-3.2079780	2.7762980	0.0004560
C	-2.7063740	0.0047710	0.0072010
C	-4.2582620	1.8956620	-0.0001440
C	-4.0433380	0.5023120	-0.0003620
C	-2.4707640	-1.4006870	-0.0035790
C	-5.1246610	-0.4134950	0.0001720
C	-3.5750110	-2.2559550	-0.0013640
C	-4.8854270	-1.7622760	-0.0009470
C	2.1291640	0.8964460	0.0070610
C	-0.0297460	-0.9505500	-0.0061190
C	2.3891730	-0.4943390	-0.0050800
C	1.3123840	-1.4157710	0.0043560
C	1.6399230	3.6578760	-0.0004870
H	-0.9803730	4.2723660	0.0056620
H	-3.3894840	3.8457090	0.0011840
H	-5.2787230	2.2637610	-0.0016580
H	-6.1383530	-0.0279530	-0.0008700
H	-3.4329740	-3.3281850	-0.0025910
H	-5.7136930	-2.4617530	-0.0005870
C	3.2215620	1.8275070	-0.0010930
C	-1.1105850	-1.8819670	0.0016430
C	3.7282250	-0.9657190	0.0004030
C	1.5855520	-2.8071520	0.0007690
C	0.4901690	-3.7089870	0.0001290
C	-0.7990490	-3.2590040	0.0010360
C	2.9190790	-3.2473120	0.0006140
H	0.6932650	-4.7746820	-0.0009700
H	-1.5983820	-3.9880180	0.0003760
C	3.9645130	-2.3479520	0.0012480
C	2.9362480	3.2007650	-0.0018150
C	4.5460370	1.3239550	-0.0009070
C	4.7940400	-0.0145880	-0.0001660
H	1.4388230	4.7238300	-0.0003510
H	3.1184600	-4.3136400	0.0004870

H	4.9887930	-2.7052510	0.0023710
H	3.7603090	3.9065430	-0.0041650
H	5.3680050	2.0322910	-0.0025560
H	5.8140320	-0.3836850	0.0004870

OO9H-S_0

C	3.1966610	2.3035520	0.4195250
C	2.3478230	3.4130340	0.3481110
C	2.6557790	1.0097830	0.2091750
C	0.9922110	3.2788380	0.0956690
C	1.2689170	0.8516090	-0.0037230
C	0.4292770	1.9779510	-0.0524110
C	0.1329070	4.4199740	-0.0281930
C	-0.9744530	1.8142840	-0.2142760
C	-1.1875950	4.2746910	-0.2589490
C	-1.7928060	2.9775960	-0.3415070
C	-1.5529800	0.5237260	-0.2460010
C	-3.1422020	2.8166470	-0.5706800
C	-2.9750150	0.4171180	-0.1423320
C	-3.7506980	1.5602330	-0.4604810
C	3.5148470	-0.1201910	0.1861640
C	0.7215400	-0.4381640	-0.2643960
C	2.9858690	-1.3989270	-0.1217000
C	1.5981010	-1.5505610	-0.3831930
C	4.5925190	2.4272720	0.6703660
H	2.7650350	4.4061410	0.4843800
H	0.5740260	5.4071610	0.0602390
H	-1.8287360	5.1432570	-0.3663160
H	-3.7619820	3.6844560	-0.7735560
C	-3.6869660	-0.7435250	0.3555220
C	-5.1629850	1.4349100	-0.6369870
C	4.8957240	0.0349690	0.4264400
C	-0.6650140	-0.6008850	-0.4545950
C	3.8464730	-2.5165390	-0.1935290
C	1.0946290	-2.8114650	-0.7672150
C	-0.2732090	-2.9111910	-1.0977860
C	-1.1153350	-1.8464750	-0.9533860
C	1.9811260	-3.9159520	-0.8433670
H	-0.6545160	-3.8529790	-1.4781320
H	-2.1552030	-1.9559580	-1.2268830
C	3.3046410	-3.7769200	-0.5545570
C	5.4047710	1.3376500	0.6834490
C	5.7288790	-1.1052880	0.3812560
C	5.2220060	-2.3380350	0.0803980

H	5.0002990	3.4171750	0.8461560
H	1.5824200	-4.8811800	-1.1375750
H	3.9705840	-4.6316860	-0.6106610
H	6.4670210	1.4497790	0.8742390
H	6.7891100	-0.9842510	0.5775290
H	5.8772700	-3.2017300	0.0369370
C	-5.0892760	-0.8351210	0.1629660
C	-5.7971150	0.2650500	-0.4047400
C	-3.0748170	-1.7333230	1.1501220
C	-5.7843310	-1.9697580	0.6208470
C	-3.7802420	-2.8123030	1.6230270
C	-5.1421280	-2.9551010	1.3273560
H	-5.7138170	2.3089370	-0.9681860
H	-6.8659810	0.1698130	-0.5639790
H	-2.0290810	-1.6331840	1.4057540
H	-6.8516720	-2.0325100	0.4352710
H	-3.2791450	-3.5503880	2.2389370
H	-5.6908310	-3.8178330	1.6875320

OO9H-S$_1$

C	3.2094930	2.3076640	0.4239370
C	2.3739880	3.4098250	0.3776280
C	2.6641390	1.0059530	0.1964400
C	0.9902320	3.2866820	0.1364470
C	1.2707470	0.8538300	-0.0134700
C	0.4285760	1.9850560	-0.0242820
C	0.1416690	4.4079730	0.0322950
C	-0.9675770	1.8227120	-0.1789070
C	-1.2000310	4.2547820	-0.2223420
C	-1.7876390	2.9772100	-0.3274910
C	-1.5425700	0.5242100	-0.2487300
C	-3.1545940	2.8000400	-0.6237290
C	-2.9875510	0.4138200	-0.1618140
C	-3.7597930	1.5563180	-0.5358700
C	3.5097240	-0.1183930	0.1579310
C	0.7233460	-0.4224030	-0.2942900
C	2.9706510	-1.3969890	-0.1418110
C	1.5836950	-1.5421300	-0.3909580
C	4.6156480	2.4187530	0.6652800
H	2.7927630	4.4015840	0.5183000
H	0.5676780	5.3996630	0.1391850
H	-1.8330300	5.1277660	-0.3415470
H	-3.7618930	3.6703010	-0.8523800
C	-3.6713500	-0.7074940	0.3766910

C	-5.1721270	1.4092110	-0.7224100
C	4.9072230	0.0264810	0.3930870
C	-0.6815430	-0.5783090	-0.4743250
C	3.8257960	-2.5237140	-0.2065570
C	1.0615300	-2.8156290	-0.7461740
C	-0.3163090	-2.9151180	-1.0632580
C	-1.1525720	-1.8505890	-0.9395970
C	1.9357590	-3.9180160	-0.8135450
H	-0.6998900	-3.8640120	-1.4234780
H	-2.1902480	-1.9467790	-1.2258020
C	3.2710920	-3.7828650	-0.5425140
C	5.4222530	1.3272230	0.6566060
C	5.7244580	-1.1124040	0.3427570
C	5.2028560	-2.3507030	0.0518070
H	5.0286430	3.4044250	0.8520540
H	1.5309020	-4.8869860	-1.0866530
H	3.9285990	-4.6442180	-0.5943070
H	6.4863590	1.4327800	0.8407150
H	6.7876840	-1.0018110	0.5287440
H	5.8537880	-3.2174510	0.0068270
C	-5.0886710	-0.8196760	0.1999440
C	-5.8005180	0.2530780	-0.4131520
C	-3.0282630	-1.7093470	1.1559380
C	-5.7565820	-1.9555770	0.6679370
C	-3.7206780	-2.7955180	1.6297970
C	-5.0868530	-2.9442500	1.3603920
H	-5.7281560	2.2580680	-1.1064710
H	-6.8687920	0.1439900	-0.5684810
H	-1.9862110	-1.5851730	1.4156940
H	-6.8258640	-2.0347290	0.5001550
H	-3.2054080	-3.5357700	2.2316260
H	-5.6228460	-3.8118190	1.7276930

8. Reference

1. Yanai, T.; Tew, D.P.; Handy, N.C. A New Hybrid Exchange–Correlation Functional Using the Coulomb-Attenuating Method (CAM-B3LYP). *Chem. Phys. Lett.* **2004**, *393*, 51–57.
2. Grimme, S. Density functional theory with London dispersion corrections. *Wiley Interdiscip. Rev. Comput. Mol. Sci.* **2011**, *1*, 211–228.
3. Adamo, C.; Barone, V. Toward Reliable Density Functional Methods without Adjustable Parameters: The PBE0Model. *J. Chem. Phys.* **1999**, *110*, 6158–6170.
4. Zhao, Y.; Truhlar, D.G. The M06 Suite of Density Functionals for Main Group Thermochemistry, Thermochemical Kinetics, Noncovalent Interactions, Excited States, and Transition Elements: Two New Functionals and Systematic Testing of Four M06-Class Functionals and 12 Other Functionals. *Theor. Chem. Acc.* **2008**, *120*, 215–241.
5. Chai, J.-D.; Head-Gordon, M. Long-Range Corrected Hybrid Density Functionals with Damped Atom–Atom Dispersion Corrections. *Phys. Chem. Chem. Phys.* **2008**, *10*, 6615–6620.
6. Weigend, F.; Ahlrichs, R. Balanced basis sets of split valence, triple zeta valence and quadruple zeta valence quality for H to Rn: Design and assessment of accuracy. *Phys. Chem. Chem. Phys.* **2005**, *7*, 3297–3305.
7. Frisch, M.J.; Trucks, G.W.; Schlegel, H.B.; Scuseria, G.E.; Robb, M.A.; Cheeseman, J.R.; Scalmani, G.; Barone, V.; Mennucci, B.; Petersson, G.A.; et al. Gaussian, version 09, Revision E.01; Gaussian Inc.: Wallingford, CT, USA, 2013.
